# Supplementary material for: Digital Kennison: a bioinformatics pipeline for rapid mapping of sequences to the Drosophila melanogaster Y chromosome
Source: G3 (Bethesda). 2026 Sep 10;16(11):jkag253. doi: 10.1093/g3journal/jkag253 (PMC13732432; doi:10.1093/g3journal/jkag253)
Supplement: Supplementary file 1 [file jkag253_supplementary_data.pdf]

File S1 - Supplementary Material

Digital Kennison: A bioinformatics pipeline for rapid mapping of sequences to the *Drosophila melanogaster* Y chromosome

Fabiana Uno, A. Bernardo Carvalho

Contents

S1. Supplementary Figures 5
Figure S1 5
Figure S2. 7
Figure S3. 9
S2. Supplementary Tables 10
Table S1 10
Table S2 11
Table S3.. 11
Table S4. 12
Table S5. 12
Table S6. 13
Table S7. 14
S3. Supplementary Methods 14
S3.1. Identifying Y-linked sequences. 14
S.3.2. Y-linked scaffold extraction and database construction 15
S3.3. The pipeline 16
Figure S4. 17
S3.3.1. *Identity-threshold selection and occupancy scoring* 17
S3.3.2. *Pre-filtering rules* 18
S3.3.3. *Contamination correction* 19
S3.3.4. *Boundary-spanning detection* 19
S3.3.5. *Tiered outlier detection* 20
S3.3.6. *Centromere detection* 22
S3.3.7. *Assembly-aware classification* 22
S3.3.8. *Confidence score calculation* 23
S3.3.9. *Read-based validation* 25
S3.3.10. *Masking-aware occupancy calculation* 26
S3.3.11. *Command-line parameters* 27

S3.4. Cross-assembly localization of FlyBase-annotated *Su(Ste)* sequences 29

Table S8. 29

S3.5. Application to external Supplementary Datasets 29

S3.5.1. Classification and Cross-Assembly Validation of Unlocalized R6 Scaffolds 29

S3.5.2. Classification of de novo genome assemblies: Windowed composition analysis. 31

S3.5.3. Application to recently transferred Y-linked sequences (Tobler et al. 2017) 32

Table S9 32

S3.6. Auxiliary scripts 34

S4.1. Evaluation of Classification Accuracy 35

S4.1.1. *Single-Copy Gene Set* 35

Table S10 36

S4.1.2. *Mst77Y Gene Cluster* 36

Table S11. Summary of classification accuracy for Mst77Y gene family relative to known chromosomal locations across test runs 37

Table S12. 38

S4.1.3. *Y-Linked Non-Coding RNA Set* 38

Table S13. Digital Kennison classification accuracy for Y-linked non-coding RNA sequences across parameter settings. 40

Table S14. 41

S4.1.4. *Cross-assembly evidence for kl-1/centromere placement of three FlyBase-annotated Su(Ste) sequences* 41

S4.1.5. *Cross assembly analysis of CR40629-RC* 42

Table S15. 43

S4.2. Centromere contig classification 44

S4.3. Assessment of classification confidence 46

Figure S5. 46

Table S16 47

S4.4. Cross-assembly evidence for revised chromosomal assignment of five R6 minor scaffolds 47

Table S17. 48

Table S18 49

Table S19 50

S4.5. Classification of unlocalized scaffolds in de novo genome assemblies 50

S4.6. Localizing sequences recently transferred to the Y chromosome – Tobler et al. (2017)..... 56

Table S24. 58

S5. References **58**

S1. Supplementary Figures

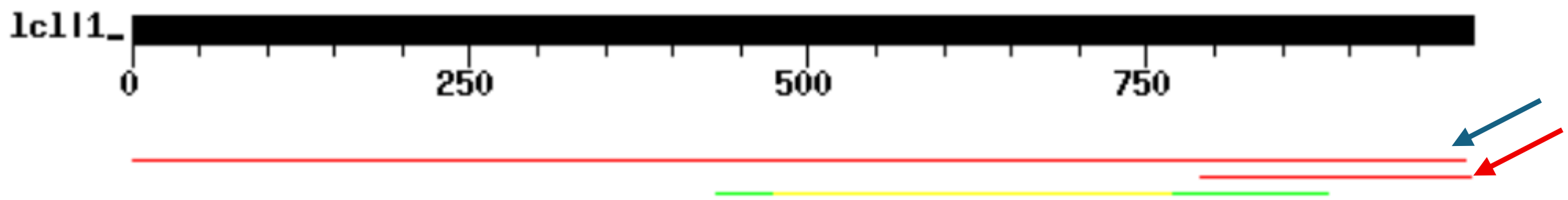

Figure S1. BLAST+ blastn search of the *FDY* coding sequence against the kl-5⁻ database. The search recovered both genuine *FDY* hits (99% identity, ~50–200 bp alignments, red arrow) and multiple high-identity hits to its autosomal paralog *vig2* (93–99% identity, ~100–470 bp alignments, blue arrow).

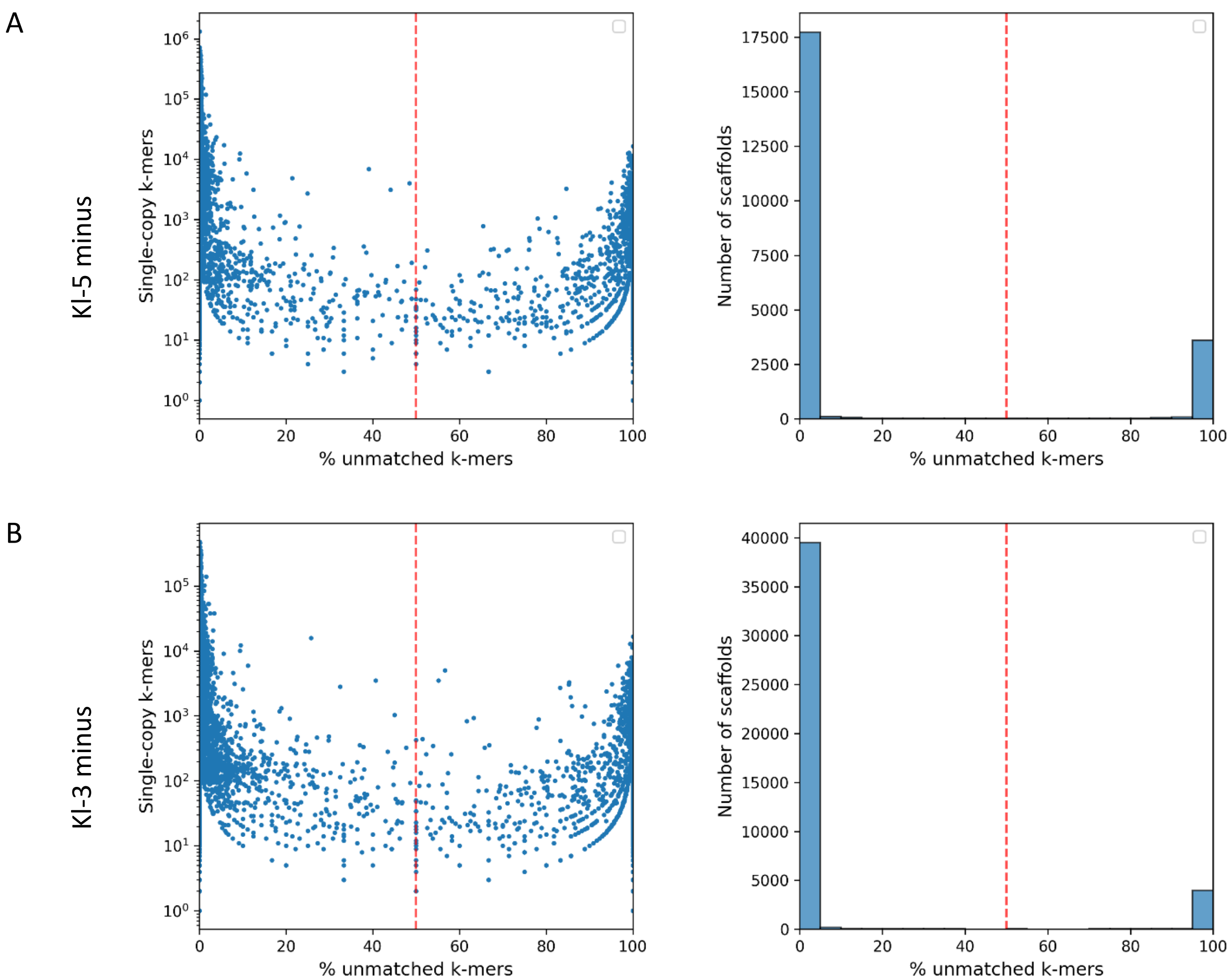

C

Kl-2 minus

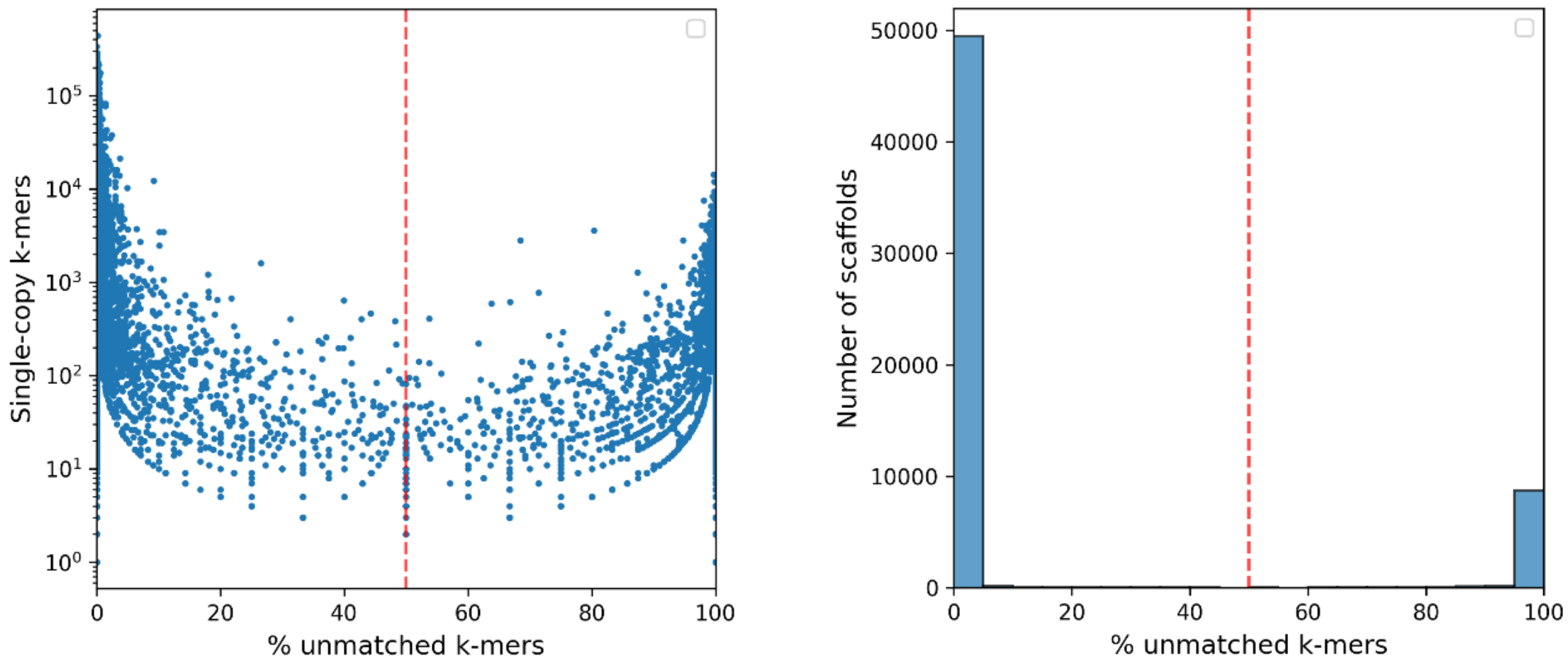

D

Kl-1 minus

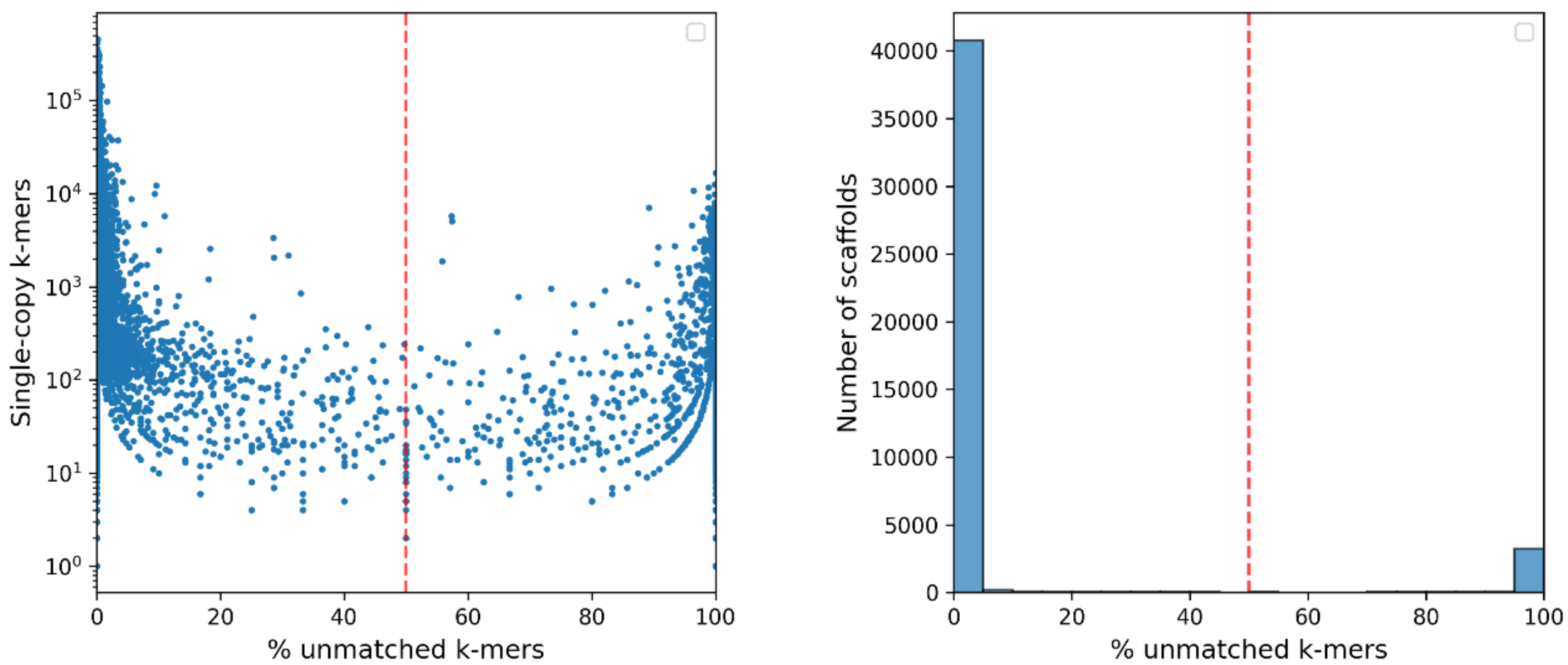

E

Ks-1 minus

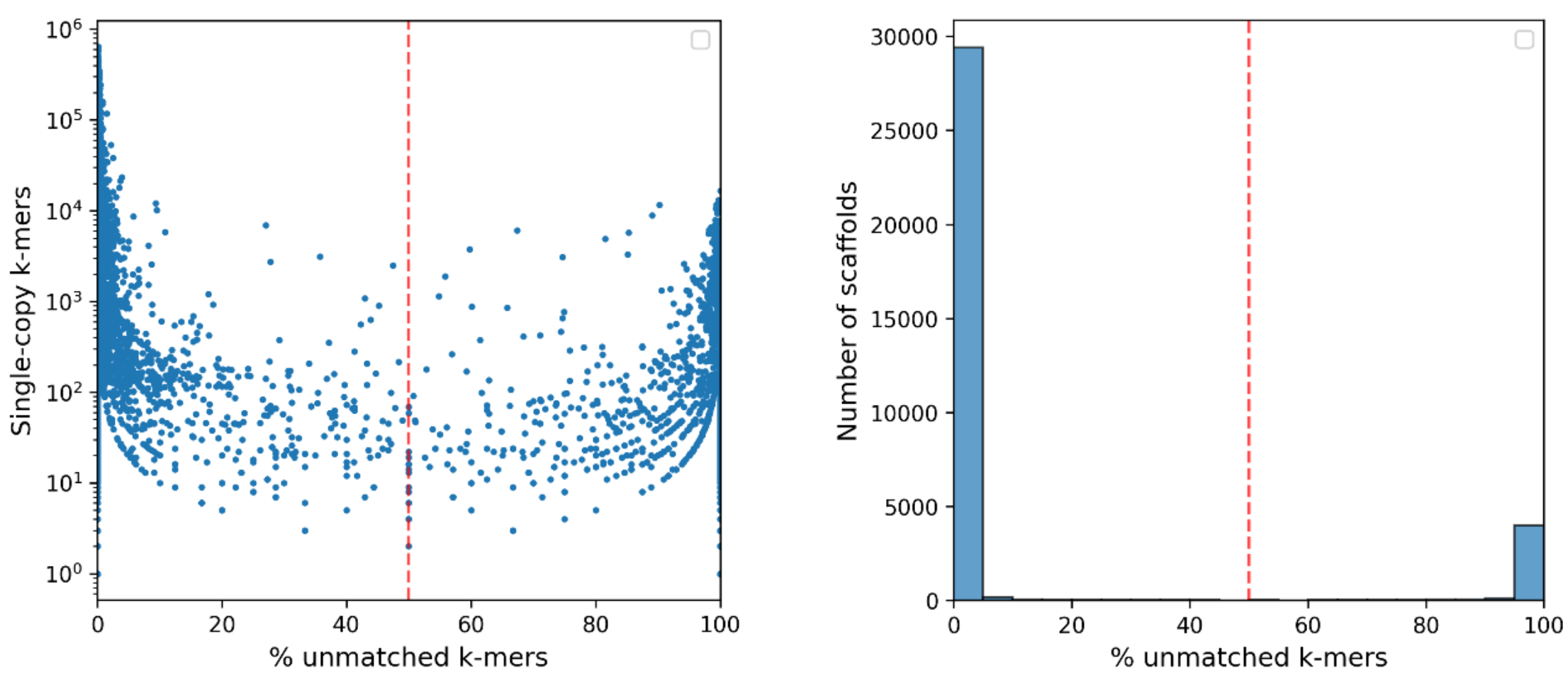

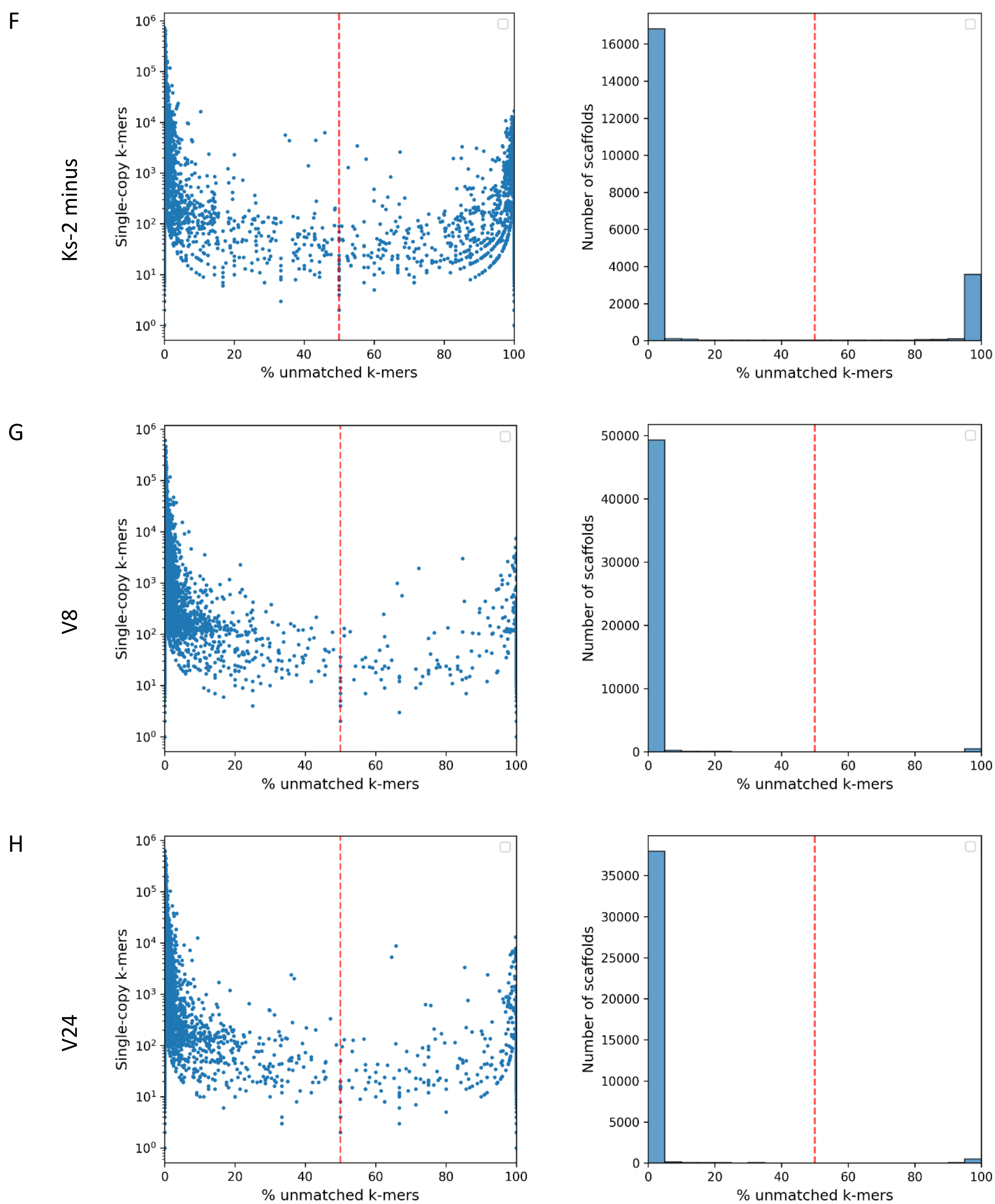

Figure S2. YGS (Y-chromosome Genome Scan) analysis of translocation strain assemblies used in the Digital Kennison pipeline. Each panel shows the distribution of PVSC_UK values (Percentage of Valid Single-Copy k-mers Unmatched by female reads) across assembled contigs for a given strain. Scaffolds

with PVSC_UK ≥ 50% were considered Y-linked and were retained for database construction. Non-informative scaffolds (less than 20 single-copy k-mers) were excluded from the analysis. The full YGS output tables are available in *Supplementary Dataset 1* (*sheets S1-S7*).

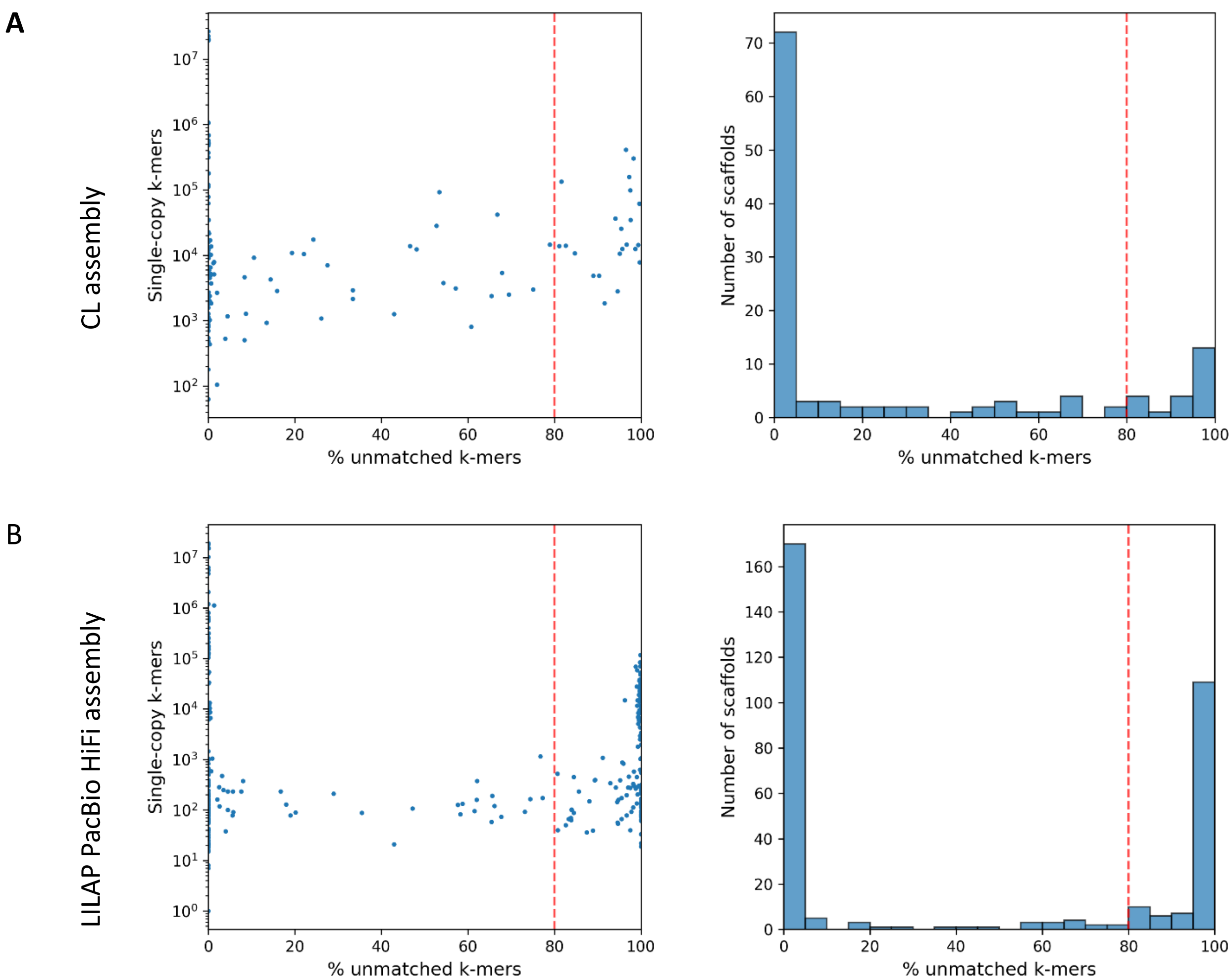

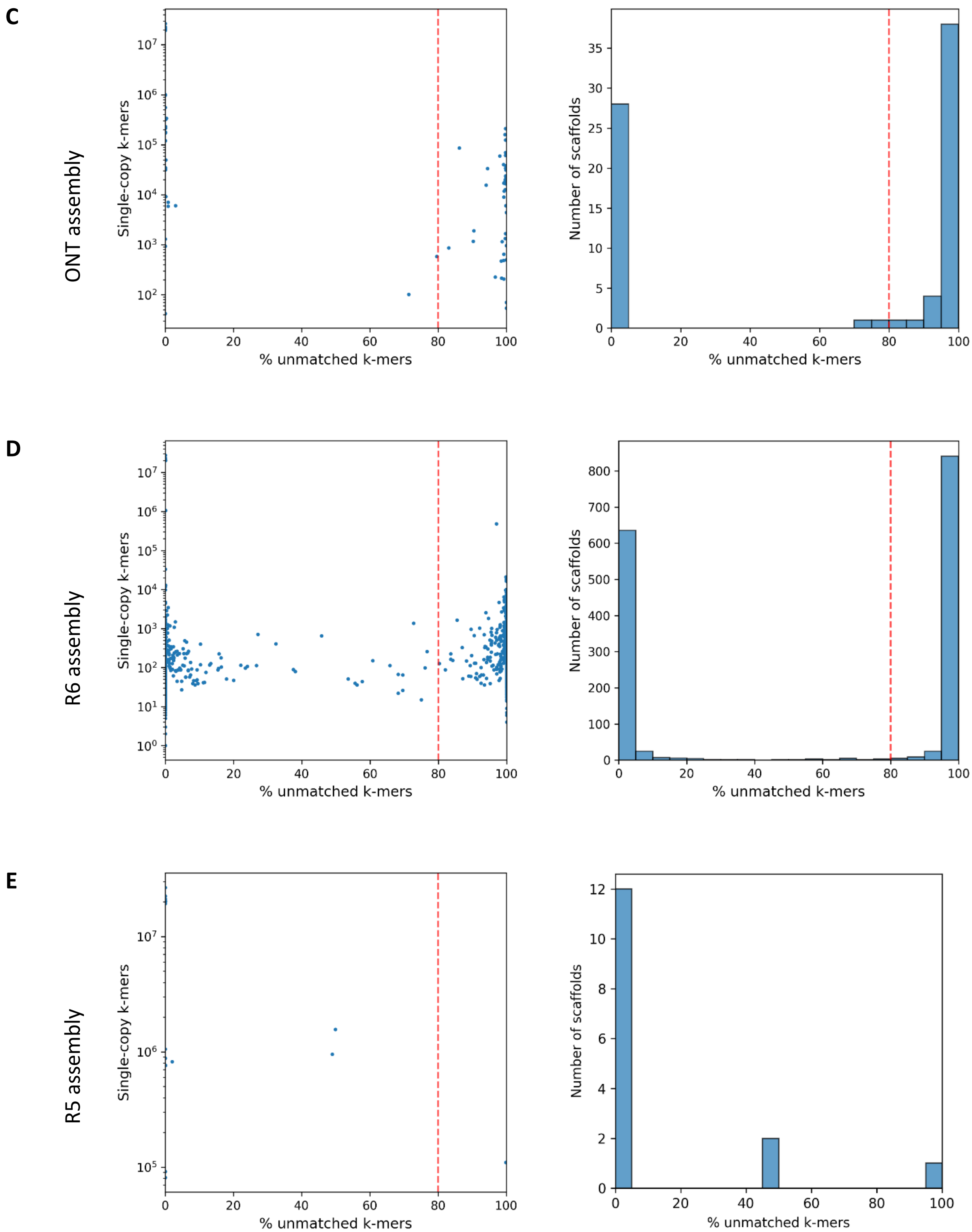

Figure S3. Distribution of P_VSC_UK values across five *D. melanogaster* assemblies used to validate Y-linkage assignments of unlocalized R6 scaffolds. Each panel shows a scatter plot (right panel) and a

histogram (left panel) of P_VSC_UK values (percentage of valid single-copy k-mers unmatched by female reads): (A) CL — PacBio assembly (Chang and Larracuente 2019); (B) LILAP — PacBio HiFi assembly (reads from Jia et al. 2024); (C) ONT — Nanopore assembly (reads from Kim et al. 2024)); (D) R6 — Release 6 reference assembly; (E) R5 — Release 5 reference assembly. YGS analyses were performed at k = 21. Scaffolds containing fewer than 20 single-copy k-mers were excluded as non-informative (Carvalho and Clark 2013); scaffolds with P_VSC_UK ≥ 80% (vertical dashed line) were classified as Y-linked. Per-scaffold P_VSC_UK values are provided in *Supplementary Dataset 1* (*sheet S8-S12*).

S2. Supplementary Tables

Table S1. Translocation strains used in this study, originally described by Kennison (1981). Strain details and Bloomington stock numbers from Carvalho et al. (2015).

Strain full name	Abridged name	Bloomington ID	Y breakpoint*
Oregon-R	ORE-R	-	-
y[1]; cn[1] bw[1] sp[1]	ISO1	2057	-
T(1;Y)V24, y[1] w[1] f[1]: y[+] B[S]/C(1)A, y[1]	V24	3060	h4
T(1;Y;3)W27, y[1] y[+] w[1] f[1] B[S] / C(1)A, y[1]	W27	4049	h9-h10
T(1;Y)E15, y[1] w[1]: y[+] B[S]/C(1)A, y[1]	E15	3224	h12-h13
T(1;Y)F12, y[1] w[1] f[1]: y[+] B[S]/C(1)A, y[1]	F12	3158	h15
T(1;Y)W19, y[1] w[1] f[1]: y[+] B[S]/C(1)A, y[1]	W19	2950	h20-h21
T(1;Y)V8, y[1] w[1]: y[+] B[S]/C(1)A, y[1]	V8	478	h24

* Breakpoint locations obtained from FlyBase. Breakpoints in the middle of a band appear as "h4" and so forth; those between two bands appear as "h9-h10".

Table S2. Crosses used to generate males lacking defined regions of the Y chromosome, originally described by Kennison (1981). Strains and DNA samples are those used by Carvalho et al. (2015), with the addition of a "Missing fertility region (s)" column to indicate the Y-chromosome region absent in each tested genotype.

Cross (♀ × ♂)	Tested genotype*	Abridged name	Missing bands	Missing fertility region(s)
V24 × V24 †	X^D Y^P V24 / 0	kl-5 $^-$	h1 - h4§	kl-5
V24 × W27	X^D Y^P W27 / Y^D X^P V24	kl-3 $^-$	h4§ - h9	Kl-3
W27 × E15	X^D Y^P E15 / Y^D X^P W27	kl-2 $^-$	h10 - h12	Kl-2
E15 × F12	X^D Y^P F12 / Y^D X^P E15	kl-1 $^-$	h13 - h15§	Kl-1
V8 × W19	X^D Y^P W19 / Y^D X^P V8	ks-1 $^-$	h21 - h24§	Ks-1
V8 × V8 ‡	X^D Y^P V8/ 0	ks-2 $^-$	h24§ - h25	Ks-2
ISO1 × V8	Y^D X^P V8 / X	Y^D X^P V8	h1 - h24§	all except ks-2
ORE-R × V24	Y^D X^P V24 / X	Y^D X^P V24	h4§ - h25	all except kl-5

* XD YP is the acentric distal portion of the X chromosome (XD) appended to the centric proximate portion of the Y chromosome (YP). The reciprocal element is called YD XP ; note that it lacks the Y centromere (1).

† Exceptional X/0 males, identified as w B+ males

‡ Exceptional X/0 males, identified as w B y males

§ Breakpoints in the middle of the band

Table S3. Sequencing depth and de novo assembly metrics for the eight translocation-strain genomes used to construct the Digital Kennison BLAST databases. Reads were trimmed with TrimGalore and assembled with SPAdes; assembly completeness was assessed with BUSCO (Diptera_odb10).

Strain	Read pairs (M)	Coverage* (x)	Scaffolds (n)	N50 (Mb)	Largest scaffold (Mb)	auN†	Total assembly (Mb)	BUSCO (C)%
kl-5$^-$	35.8	60	157731	0.05	0.46	0.09	120.97	99.1
kl-3$^-$	37.7	63	953543	0.03	0.44	0.05	120.87	97.7
kl-2$^-$	41.1	68	146682	0.06	0.49	0.09	121.21	96.8
kl-1$^-$	33.3	55	91407	0.17	1.34	0.21	120.63	97.4
ks-1$^-$	39.3	65	138492	0.1	0.64	0.15	121.1	98.3
ks-2$^-$	34.3	57	89190	0.19	0.73	0.22	120.73	99.3
V24	37.0	62	135558	0.07	0.63	0.1	120.49	97.7
V8	37.2	62	164622	0.04	0.62	0.08	120.5	96.2

*Coverage estimated as (read pairs × 2 × 150 bp) / 180 Mb (assumed *D. melanogaster* genome size).

†Area under the curve for scaffold length distribution (higher values indicate more contiguous assemblies)

Table S4. Read database preparation for the read-based classification module of the Digital Kennison pipeline. For each strain, paired-end reads were aligned to the *D. melanogaster* R6 reference (FlyBase release 6.64) with minimap2; reads mapping to autosomes (2L, 2R, 3L, 3R, 4) or the X chromosome were excluded by read name to suppress signal from autosomal paralogs. Retained read pairs comprise those mapping to the Y chromosome, to unplaced or unlocalized contigs, and unmapped reads; mitochondrial reads were also retained.

Strain	Read pairs (M)	Reads pairs retained after prefiltering (M)	% retained	% autosomal/X removed
kl-5$^-$	35.8	5.0	14.1	85.9
kl-3$^-$	37.7	4.2	11.2	88.8
kl-2$^-$	41.1	7.8	19.0	81.0
kl-1$^-$	33.3	4.3	13.1	86.9
ks-1$^-$	39.3	5.4	13.9	86.1
ks-2$^-$	34.3	4.2	12.4	87.6
V24	37.0	3.4	9.3	90.7
V8	37.2	3.9	10.6	89.4

Table S5. Digital Kennison pipeline parameter optimization across three Y-linked sequence categories: single-copy genes, Mst77Y cluster, and ncRNA loci.

Supplementary Dataset	Run	Hit weight	Strict identity	Assembly-aware	Y-linked	Permissive thresholds	Scaffold-aware	read validation
Protein-coding, single copy	Run 1	default (2.0)	N	N	N	N	N	N
	Run 2	default (2.0)	Y	N	N	N	N	N
	Run 3	default (2.0)	Y	Y	N	N	N	N
	Run 4	default (2.0)	Y	Y	Y	N	N	N
	Run 5	default (2.0)	Y	Y	Y	Y	N	N
	Run 6	default (2.0)	Y	Y	Y	Y	Y	N
	Run 7	default (2.0)	Y	Y	Y	Y	Y	Y
Multiple copies (Mst-77Y cluster)	Run 8	default (2.0)	N	N	N	N	N	N
	Run 9	default (2.0)	Y	N	N	N	N	N
	Run 10	default (2.0)	Y	Y	N	N	N	N
	Run 11	default (2.0)	Y	Y	Y	N	N	N
	Run 12	default (2.0)	Y	Y	Y	Y	N	N
	Run 13	default (2.0)	Y	Y	Y	Y	Y	N
	Run 14	default (2.0)	Y	Y	Y	Y	Y	Y
ncRNA loci	Run 15	default (2.0)	N	N	N	N	N	N
	Run 16	default (2.0)	Y	N	N	N	N	N
	Run 17	default (2.0)	Y	Y	N	N	N	N
	Run 18	default (2.0)	Y	Y	Y	N	N	N
	Run 19	default (2.0)	Y	Y	Y	Y	N	N
	Run 20	default (2.0)	Y	Y	Y	Y	Y	N
	Run 21	default (2.0)	Y	Y	Y	Y	Y	Y
	Run 22	5	Y	Y	Y	Y	Y	Y

Table S6. Y-linked genes used as a benchmark for pipeline validation, with primary cytological localization references. This table summarizes the cytological positions, fertility region assignments, mapping method, and key references for well-characterized Y-linked genes, including protein-coding and repetitive elements. Direct cytological mapping (FISH, BAC-FISH, or Y-deficiency Southern) localized only *kl-5/Dhc-Yh3* to h3 (Gepner and Hays 1993), *Mst77Y* genes to h18–h19 (Russell and Kaiser 1993), *Pp1-Y2* to h18 (Abad et al. 2004), and *FDY* to the interval h15–h20 (Carvalho et al. 2015). All other h-band assignments are inferred from the correspondence between fertility regions and cytological bands defined by Kennison (1981) translocation breakpoints (see Table S2) and the cytogenetic map of (Gatti and Pimpinelli 1983).

gene	Cytological location	Fertility region	Detection assay	References
Pp1-Y1	h1-h4§	kl-5	PCR (Kennison X-Y translocations)	(Carvalho et al. 2001)
Kl-5	h3	kl-5	Southern blot (Kennison X-Y translocations + Y deficiencies)	(Gepner and Hays 1993)
PRY	h1-h9	kl-5/kl-3	PCR (Kennison X-Y translocations)	(Carvalho et al. 2001)
kl-3	h4§ - h9	kl-3	PCR (Kennison X-Y translocations)	(Carvalho et al. 2000)
Kl-2	h10 - h12	kl-2	PCR (Kennison X-Y translocations)	(Carvalho et al. 2000)
ARY	h10 - h12	kl-2	PCR (Kennison X-Y translocations)	(Vibranovski et al. 2008)
Su(Ste) canonical cluster	h11 (or h11-h13)	kl-2	Southern blot (Kennison X-Y translocations); FISH	(Livak 1990; Bonaccorsi and Lohe 1991)
PCKR (pseudo-*CK2β* repeat)	h14–h16	kl-1/centromere	Illumina male/female read mapping + PCR validation	(Chang and Larracuente 2019)
Ppr-Y	h10- h15§	kl-2/kl-1	PCR (Kennison X-Y translocations)	(Carvalho et al. 2001)
WDY	h13 - h15§	kl-1	PCR (Kennison X-Y translocations)	(Vibranovski et al. 2008)
FDY	h15-h20	centromere	PCR (Kennison X-Y translocations)	(Krsticevic et al. 2015)
Mst77Y genes	h18-h19	centromere	Southern blot (Y deficiencies + Kennison X-Y translocations)	(Russell and Kaiser 1993)
Pp1-Y2	h18	centromere	Southern blot (Y deficiencies)	(Abad et al. 2004)
ORY	h21-h24§	ks-1	PCR (Kennison X-Y translocations)	(Carvalho et al. 2001)
CCY	h24§-h25	ks-2	PCR (Kennison X-Y translocations)	(Carvalho et al. 2001)

§ Breakpoints in the middle of the band

Table S7. Effect of the Y-linked filter over the total number of BLAST+ blastn hits obtained for the single-copy genes in each strain database

strain	Total hits	retained	Y-linked (%)
kl-5	518	383	73.9
kl-3	519	347	66.9
kl-2	1133	531	46.9
kl-1	540	407	75.4
ks-1	567	379	66.8
ks-2	557	368	66.1
V8	47	3	6.4
V24	67	24	35.8

S3. Supplementary Methods

S3.1. Identifying Y-linked sequences.

The YGS (Y-chromosome Genome Scan, Carvalho & Clark, 2013) method identifies Y-linked sequences by comparing assembled genome scaffolds against female sequencing reads using k-mer analysis. The method takes as input: (1) female short sequencing reads; (2) assembled male or mixed-sex genome scaffolds from the organism of interest; and optionally (3) male Illumina reads for error correction of assemblies. The output includes the PVSC_UK value for each scaffold, defined as the *Percentage of Valid Single-Copy k-mers Unmatched by female reads*.

The YGS method was applied using a k-mer size of 24 for the Illumina-based translocation-strain assemblies, with contigs classified as Y-linked at P_VSC_UK ≥ 50%. For long-read assemblies (R6, CL, LILAP, and ONT), a k-mer size of 21 and a more stringent threshold (P_VSC_UK ≥ 80%) were used, reflecting their higher contiguity and improved separation between Y-linked and autosomal/X-linked sequences in YGS profiles.

Scaffolds with fewer than 20 single-copy k-mers were considered non-informative and excluded from the analysis.

Input data

Male genome assemblies were produced as described above (Genome sequencing and database construction). Male Illumina reads for the *D. melanogaster* Iso1 strain were obtained from two NCBI BioProjects: PRJEB8008 (accessions ERR701706–ERR701711) and PRJNA1227112 (accession SRR3245668).

Genomic DNA for the female Illumina dataset was extracted from 40 freshly collected Iso1 females using the Promega Wizard Genomic DNA Purification Kit (Cat. #A1120). Library preparation and sequencing were performed by Macrogen (Korea) using the Illumina TruSeq Nano DNA PCR-free protocol, generating 151 bp paired-end reads with an average insert size of 350 bp. The newly generated reads have been deposited in the NCBI Sequence Read Archive (accession SRR39563608). These reads were combined with two publicly available, high-quality Iso1 Illumina datasets (accession numbers SRR11460799 and SRR11906527).

Parameter Settings

YGS was run using default parameters. To increase the accuracy of Y-linkage inference, we removed likely error-derived *k-mers* as recommended in Carvalho and Clark (2013). A PVSC_UK threshold of ≥50% was used to conservatively define Y-linked scaffolds and reduce false positives. The YGS output for all translocation strain assemblies is available as part of *Supplementary Dataset 1* (*sheets S1-S7*), and corresponding plots are shown in *Supplementary Figure S2*.

S.3.2. Y-linked scaffold extraction and database construction

To reduce spurious alignments to autosomal paralogs and improve the specificity of chromosomal classification, we generated a set of Y-linked scaffold identifier lists (gi_list files, one per translocation strain; Zenodo: 10.5281/zenodo.21296560) using the YGS method output as a guide. For each strain, we parsed the YGS output to retrieve contig identifiers associated with high PVSC_UK values (percentage of valid single-copy k-mers unmatched by female reads), which indicate Y-linkage. At runtime, the pipeline restricts BLAST searches against the eight deficiency-panel databases to these Y-linked scaffold sets when the `-y_linked` option is enabled (default); the databases themselves remain unmodified (see *Supplementary Results* and Supplementary Table S7).

S3.3. The pipeline

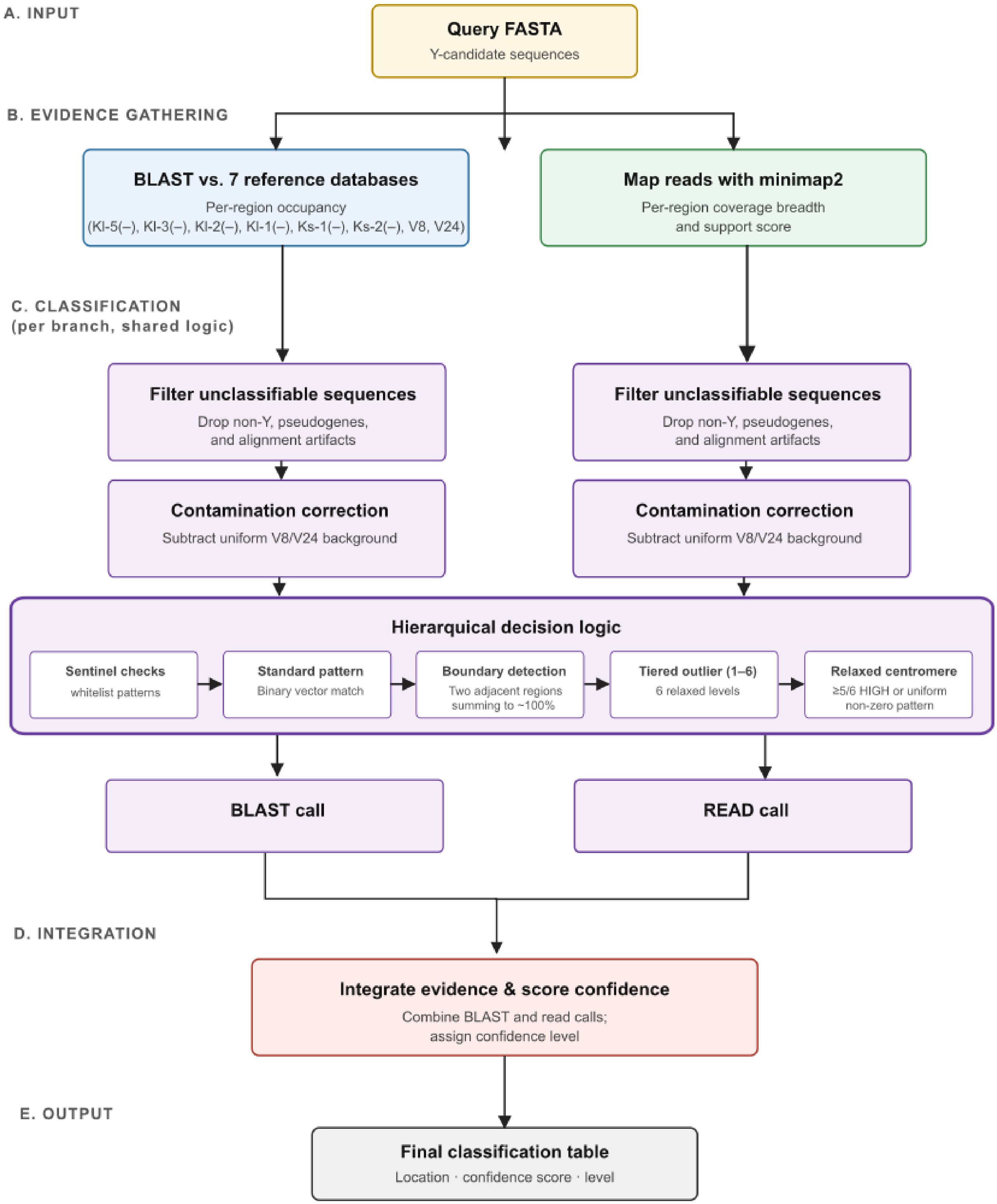

Figure S4. Overview of the Digital Kennison pipeline**.** Y-candidate sequences (A) are evaluated through two parallel evidence streams (B): (i) BLAST searches against six fertility-region subtraction databases (kl-5⁻, kl-3⁻, kl-2⁻, kl-1⁻, ks-1⁻, ks-2⁻) and two composite heterochromatic datasets (V8, V24), generating per-region occupancy and hit counts; and (ii) read alignment with minimap2 (≥99.0% identity, best hit retained), yielding coverage breadth and a support score. Both evidence streams are processed through a shared classification framework (C). Sequences are first filtered to remove non–Y-linked signatures, pseudogene-like diffuse signals, and single-database alignment artifacts; when available, YGS-derived P_VSC_UK values provide an additional filter against autosomal or X-linked contamination. Background signal in V8 and V24, attributable to shared paralogous content, is then subtracted to produce corrected occupancy profiles. Classification proceeds via a hierarchical decision scheme in which the first satisfied rule determines the assignment: (i) sentinel checks for characteristic whitelist patterns (e.g., centromere, ks-2, kl-5); (ii) direct matching to expected binary presence/absence vectors for each fertility region; (iii) boundary detection for sequences spanning adjacent regions with combined occupancy ≈100%; (iv) six progressively relaxed outlier tiers that identify the lowest-occupancy region based on gap statistics, coefficient of variation, and threshold criteria; and (v) a relaxed centromere rule for profiles with ≥5/6 regions showing high or uniformly non-zero occupancy. Sequences failing all criteria are classified as Undetermined. Independent BLAST- and read-based assignments are then reconciled (D), and a confidence score is computed. Scores are calibrated to expected accuracy. The final output (E) is a classification table reporting inferred chromosomal location, BLAST and integrated confidence scores, and a categorical confidence level (High, Medium, Low, Very Low) for each sequence.

S3.3.1. *Identity-threshold selection and occupancy scoring*

For each query, the pipeline computes BLAST occupancy across a range of identity thresholds (95.0–100.0% by default; 98.0–100.0% under `--strict_identity`). A single fixed threshold is unsatisfactory: a too-stringent value discards legitimate divergent matches, while a too-permissive value admits paralogous alignments. The pipeline therefore selects, per query, the threshold that maximizes a hit-penalized occupancy score:

occupancy score = total occupancy − (hit-weight factor × number of hits)

The hit-weight factor (default 2.0, set by `--hit_weight_factor`) down-weights excessive alignments characteristic of repetitive sequences and multi-copy gene families: a sequence aligning once at 90% occupancy is scored above one aligning ten times at 95% occupancy. Setting the factor to 0 removes the penalty entirely.

S3.3.2. *Pre-filtering rules*

Before attempting chromosomal assignment, the pipeline applies a series of pre-filtering rules to identify sequences that are unlikely to yield reliable classifications. These rules serve as quality control, flagging sequences with insufficient or anomalous occupancy patterns before they enter the main classification logic.

If YGS data is available, the pipeline first checks the P_VSC_UK value for each sequence. Sequences with P_VSC_UK below 50% are flagged as likely autosomal or X-linked contamination and excluded from further classification.

The pipeline then evaluates the overall occupancy profile across all eight databases. Sequences with zero occupancy across all databases are flagged as lacking a detectable Y-linked signal in the current assemblies. Sequences showing uniform low occupancy (defined as maximum occupancy below 60% with a range below 20% across databases) are flagged as likely pseudogenes or highly degraded copies whose regional signal cannot be resolved. When P_VSC_UK data is available, this classification is refined: sequences with high P_VSC_UK (≥90%) but uniformly low occupancy are annotated as likely true Y-linked sequences whose regional assignment is limited by assembly fragmentation or alignment issues, rather than by sequence degradation.

Additional filters address sparse occupancy patterns. Sequences with zero occupancy in four or more databases (six or more in scaffold mode) are flagged as possible partial duplications. Sequences with total occupancy below 60% combined with widespread very low values are flagged as possible alignment artifacts. Sequences showing occupancy in only a single database while all others are at or near zero are similarly flagged.

The pipeline operates in two modes with respect to these thresholds. In standard (gene) mode, thresholds are relatively strict, appropriate for well-defined coding sequences. In scaffold mode (enabled with the `--scaffolds` flag), thresholds are relaxed and adapted to sequence length, accommodating the

broader and more variable occupancy patterns expected from long genomic scaffolds that may span multiple fertility regions.

Sequences flagged by any pre-filtering rule receive an "Undetermined" classification with a descriptive annotation indicating the likely cause. Only sequences that pass all pre-filtering checks proceed to the main classification logic.

S3.3.3. *Contamination correction*

Sequences that pass pre-filtering enter the main classification logic, which begins with a contamination correction step. In some cases, autosomal or X-linked paralogs produce a uniform background signal across all databases, including V8 and V24. Because genuine Y-linked sequences are expected to be absent from at least one database (and V8 and V24 should show complementary patterns for most regions), elevated and similar occupancy in both V8 and V24 can indicate paralogous contamination rather than true Y-linked signal.

The pipeline detects this pattern when both V8 and V24 occupancy exceed the absence threshold, and their values are within 15 percentage points of each other. Before applying any correction, the algorithm checks whether the V8 or V24 signal follows an expected Y-linked pattern — specifically, whether V8 is elevated because the sequence genuinely maps to the ks-2 region (high V8, low ks-2^-, other regions high), or V24 is elevated because it maps to kl-5 (high V24, low kl-5^-, other regions high). These "whitelist" patterns are protected from correction.

When uniform contamination is confirmed and no whitelist pattern applies, the pipeline estimates the background level as the average of V8 and V24 occupancy and subtracts this value from all databases. After subtraction, if the maximum occupancy across the six fertility databases drops below 15%, the sequence is classified as non-Y (i.e., the entire signal was paralogous). If the residual signal remains above this threshold, the corrected values are passed to the subsequent classification steps.

This correction is applied identically to both BLAST-based occupancy values and, when enabled, read-based coverage breadth values.

S3.3.4. *Boundary-spanning detection*

Some Y-linked sequences span the boundary between two adjacent fertility regions. In such cases, deletion of either region leaves part of the sequence intact, resulting in intermediate occupancy values in both corresponding databases rather than a clear presence/absence pattern. The pipeline identifies these cases and assigns them to the pair of regions they span.

Boundary-spanning detection is applied when standard pattern matching fails and proceeds according to three criteria:

i. *Intermediate-occupancy pattern*: The pipeline scans the six fertility databases and identifies regions with intermediate occupancy (above the absence threshold but below the adjusted presence threshold). Candidate sequences must show exactly two such intermediate values; all other cases are passed to subsequent classification steps.

ii. *Adjacency constraint*: The two intermediate regions must be adjacent in the cytological map (kl-5 → kl-3 → kl-2 → kl-1 → centromere → ks-1 → ks-2). Only pairs differing by one positional step are accepted. Boundaries involving the centromere (kl-1/centromere and centromere/ks-1) are handled separately by the centromere-detection module.

iii. *Sum-of-occupancy criterion:* For true boundary-spanning sequences, the two intermediate values should together approximate full coverage. The pipeline therefore requires their sum to fall within a defined window (default: 80–110%). When contamination correction is applied, this window is shifted downward to account for uniform signal subtraction (see *Supplementary Methods*).

Sequences satisfying all three criteria are classified as spanning the corresponding region pair (e.g., *kl-3/kl-2*). These assignments incur a 10-point reduction in confidence, reflecting the inherent ambiguity of multi-region localization. Sequences that fail in any criterion proceed to subsequent classification strategies.

This detection logic is applied identically to BLAST-based occupancy values and, when enabled, to read-based coverage profiles.

S3.3.5. *Tiered outlier detection*

When occupancy values do not fall cleanly into binary presence/absence categories, as often occurs with repetitive sequences, multi-copy gene families, or incompletely assembled loci, the standard pattern matching fails. To handle these cases, the pipeline employs a tiered outlier detection system that

identifies the most likely fertility region based on relative differences among the six fertility databases rather than absolute thresholds.

The logic is as follows: if a sequence is genuinely located in a specific fertility region, it should be absent (or reduced) in the corresponding deletion database while present in the others. Even when assembly fragmentation reduces all occupancy values below the standard presence threshold, the deleted region's database should still show the *lowest* value, creating a detectable outlier. The pipeline identifies this outlier and evaluates the signal strength across a series of progressively more permissive tiers.

For each sequence, the pipeline calculates the mean occupancy across all six fertility databases, identifies the database with the lowest value, and computes the gap between the mean and this minimum. It also counts how many of the remaining five databases exceed various occupancy thresholds (70%, 60%, 50%, 40%, 30%, and 20%) and, for weaker tiers, computes the coefficient of variation among the non-outlier values to assess signal uniformity.

Six tiers are evaluated in order, from most to least stringent:

i. *Tier 1 (strong outlier):* The gap exceeds 40 percentage points, the minimum is below 20%, and at least four other databases exceed 70%. This yields a high-confidence assignment.

ii. *Tier 2 (moderate outlier)*: Either the relative gap exceeds 40% of the mean with four others above 70%, or the gap exceeds 35 points with the minimum below 25% and four others above 60%.

iii. *Tier 3 (weak outlier)*: The gap exceeds 25 points, the minimum is below 30%, and at least four others exceed 50%. Assignments at this tier are flagged as requiring validation.

iv. *Tier 4 (very weak outlier)*: The minimum is below 15% and at least four others exceed 40%, with a coefficient of variation among the non-outlier values below 0.4, indicating a uniform signal despite low absolute values.

v. *Tier 5 (post-subtraction outlier)*: Designed for sequences where contamination correction has reduced all values. The minimum is below 10%; at least four others exceed 30%; the gap exceeds 15 points; and the coefficient of variation is below 0.3.

vi. *Tier 6 (extreme weak outlier)*: Only active in relaxed mode. The minimum is below 5%; at least four others exceed 20%; the coefficient of variation is below 0.25; and the mean of non-outlier values exceeds 15%. This tier captures the weakest detectable signals and is intended for highly degraded or repetitive sequences.

The pipeline applies outlier detection only when both V8 and V24 values are below 40%, thereby preventing interference from composite database signals. Classification proceeds through the

tiers in order; the first tier whose criteria are met determines the assignment and the associated confidence level.

S3.3.6. *Centromere detection*

The centromeric region presents a distinct classification pattern: sequences are expected to show high occupancy in all six fertility databases and low occupancy in both V8 and V24. However, this pattern can also arise from broad alignment of autosomal or multi-copy sequences, requiring additional safeguards.

The pipeline applies centromere detection in a hierarchical manner:

i. *Strict detection*: Sequences are classified as centromeric when all six fertility databases meet the presence threshold, and both V8 and V24 meet the absence threshold.
ii. *Relaxed detection*: When the strict pattern is disrupted (e.g., due to assembly fragmentation), centromeric classification is considered if: (i) V8 and V24 remain low; (ii) no fertility database falls below the absence threshold, and (iii) at least five fertility databases show high occupancy, or exhibit uniformly intermediate values (mean ≥50%, maximum deviation ≤15%). This captures sequences with reduced but consistent signals across fertility databases.
iii. *Contaminated centromere patterns*: In cases where paralogous signal elevates V8 or V24 above the absence threshold (up to ~40%), sequences may still be classified as centromeric if the fertility databases retain a centromeric-like pattern (no low values and at least five high or uniformly intermediate). These assignments are reported with reduced confidence.

S3.3.7. *Assembly-aware classification*

Y-linked sequences are frequently fragmented across genome assemblies due to their repetitive and heterochromatic nature. This fragmentation can produce occupancy values that fall between the presence and absence thresholds, not because the sequence is partially present in each deletion background, but because only a portion of it was successfully assembled in that strain's database. Without accounting for this, the pipeline would classify such sequences as undetermined or, worse, misidentify them as boundary-spanning genes.

When the `--assembly_aware` flag is enabled, the pipeline applies an additional classification step after the standard and outlier detection stages have failed to produce an assignment. This step first characterizes the occupancy pattern across the six fertility databases by categorizing each value as high (≥80%), intermediate (30–80%), or low (<30%).

The pipeline distinguishes three pattern types:

i. *Standard patterns* have all values clearly binary (high or low) and are handled by the normal classification logic. No assembly-aware intervention is needed.

ii. *Assembly artifact patterns* show a mix of high and intermediate values, consistent with a sequence that is present in a given deletion background but incompletely assembled. This pattern is identified when at least one fertility database shows high occupancy and at least one shows intermediate occupancy. For cases with a single intermediate value alongside multiple high values, the artifact classification is applied directly. For multiple intermediate values, the pipeline additionally checks their uniformity (coefficient of variation below 0.3), since genuine assembly fragmentation tends to produce intermediate values of similar magnitude. When an assembly artifact pattern is confirmed, the pipeline promotes intermediate values (≥30%) to presence and re-runs pattern matching against the expected presence/absence matrix, annotating the result as "assembly fragmentation suspected."

iii. *Uniform pseudogene patterns* show uniformly low occupancy across all fertility databases (maximum below 60%, range below 20%). This pattern is characteristic of autosomal or X-linked sequences (possibly a pseudogene with high similarity to a Y-linked sequence) producing false-positive BLAST hits: because such sequences are present in every strain regardless of Y-chromosome content, they generate a similar low-level signal across all databases rather than the differential pattern expected of a genuine Y-linked sequence. These cases are flagged accordingly, complementing the pre-filtering step by catching sequences that passed the initial filters with marginal values but reveal no regional signal upon closer analysis.

Complex patterns that do not fit any of the above categories receive a confidence penalty and are classified as undetermined with appropriate annotations.

S3.3.8. *Confidence score calculation*

Each classified sequence receives a confidence score (0–100) based on a weighted combination of five metrics that together capture different dimensions of classification reliability.

i. *Pattern sharpness* (40% weight) quantifies how cleanly occupancy values separate into presence and absence categories. For each database, the pipeline calculates the distance from the nearest binary value (0% or 100%) and converts this to a sharpness score where 100 represents a perfectly binary value, and 0 represents a maximally ambiguous value at 50%. The final pattern sharpness is the average across all databases. This metric carries the highest weight because a clear presence/absence separation is the most direct indicator of reliable classification.

ii. *Genomic context* (25% weight) evaluates features of the occupancy profile that are informative about classification difficulty independently of the assigned region. The score starts at 100 and is reduced by penalties for high total hit counts (indicating repetitive sequences), conflicting V8/V24 patterns (where both show elevated occupancy with a large difference between them), and multiple intermediate fertility values. A small bonus is awarded when the deficiency database returns a clear bimodal distribution with at most one intermediate value.

iii. *Identity threshold* (20% weight) reflects the stringency of the BLAST alignments supporting the classification. Higher identity thresholds yield higher scores, scaled nonlinearly: thresholds above 99.5% receive scores of 95–100, those between 98% and 99.5% receive 85–95, and those between 95% and 98% receive 70–85. Thresholds below 95% receive progressively lower scores.

iv. *Hit consistency* (15% weight) measures the concordance between the observed BLAST hit distribution and the pattern expected for the assigned region. For each region, the pipeline specifies which databases should contain hits (i.e., those in which the sequence is expected to be present). The ratio of hits in expected versus unexpected databases is computed and scaled to a 50–100 range. An additional penalty is applied when the total hit count is high (above 25 or 50 hits), as excessive hits indicate repetitive sequences where individual alignments are less informative.

v. *Classification Method Quality* - detects whether the classification used standard or relaxed rules and adds a penalty for relaxed/fallback classification methods.

After computing the weighted sum, the pipeline applies a difficulty adjustment that adds up to 8 points for sequences with predominantly binary patterns (≥85% of values clearly high or low) and subtracts up to 12 points for sequences with four or more intermediate values. Special-case penalties are then applied: undetermined classifications receive a 30–45-point reduction depending on the cause, boundary-spanning assignments receive a 10-point reduction, and assembly artifact annotations receive a 20-point reduction. When read-based validation is enabled, agreement between BLAST and read classifications adds 2–5 points to the final score.

The complete scoring function, including all thresholds and penalty values, is implemented in the pipeline source code available at the GitHub repository.

S3.3.9. *Read-based validation*

In parallel with BLAST-based occupancy analysis, the pipeline classifies sequences using prefiltered Illumina reads from each translocation strain, providing an independent line of evidence based on a fundamentally different data type. While BLAST-based classification relies on *de novo* assemblies (which may be fragmented or incomplete), read-based classification operates directly on the sequencing reads, bypassing assembly artifacts.

Read mapping and filtering: For each translocation strain, paired-end Illumina reads are mapped against the query sequences using minimap2 in short-read mode. To ensure high-confidence alignments, the pipeline retains secondary alignments during mapping but applies a strict identity filter (≥99.0% by default), requiring that the number of mismatches per alignment does not exceed 1% of the read length. When multiple alignments exist for a single read, the pipeline selects the best hit based on a hierarchical criterion: fewest mismatches, then highest mapping quality, then highest alignment score. The resulting BAM file is coordinate-sorted and indexed.

Coverage metrics: For each query sequence in each strain database, the pipeline calculates four metrics using samtools coverage: coverage breadth (percentage of query positions covered by at least one read), mean depth, mean mapping quality, and number of mapped reads. These are combined into a support score:

$$support\ score = breadth \times \sqrt{depth} \times \min(MAPQ/60, 1.0)$$

This composite metric weights breadth most heavily (as it directly mirrors the occupancy concept from BLAST-based analysis), with depth and mapping quality serving as modifiers that reflect signal strength and reliability.

Read-based classification: The classification logic mirrors the BLAST-based pipeline, using coverage breadth in place of BLAST occupancy. The same hierarchical framework is applied: sentinel checks for centromere, ks-2, and kl-5 patterns; standard pattern matching when values are binary; boundary detection for adjacent intermediate regions; tiered outlier detection (six tiers); and relaxed centromere detection. Contamination correction is applied identically, using V8 and V24 breadth values to detect and subtract uniform background signal. Presence and absence thresholds are set at 80%/10% in standard mode and 70%/20% in relaxed mode. Confidence is expressed categorically (High, Medium, Low, Very Low) based on the mean support score across fertility databases.

Integration of BLAST and read evidence: The pipeline combines the two independent classifications according to the following rules. When BLAST produces an undetermined classification but reads yield a regional assignment with at least Low confidence, the read-based result is adopted. When both methods produce assignments, agreement boosts the final confidence score (by 2–5 points depending on read confidence level), while disagreement is flagged for manual inspection. In cases of conflict, the pipeline generally defers to the method with higher confidence. When read data is insufficient or unavailable for a given sequence, the BLAST-based classification is used without modification.

S3.3.10. *Masking-aware occupancy calculation*

Genome assemblies frequently contain stretches of ambiguous bases (Ns), which represent gaps or unresolved regions, as well as soft-masked positions (lowercase bases), which flag repetitive or low-complexity sequences identified by tools such as RepeatMasker or WindowMasker. If these positions are included in the denominator when calculating occupancy, they artificially deflate the occupancy percentage: A sequence that is fully covered by BLAST alignments across all resolved positions would still report less than 100% occupancy if a portion of its length consists of Ns.

To avoid this bias, the pipeline excludes both hard-masked (N) and soft-masked (lowercase) positions from the occupancy calculation. When a FASTA query file is provided, the pipeline scans each

sequence and identifies all positions that are either N/n or lowercase, storing them as a single sorted list. During occupancy calculation, BLAST alignment coordinates are merged into non-overlapping intervals (to avoid double-counting overlapping hits), and covered positions that fall on any masked position are subtracted from the count. The denominator is adjusted to reflect only the usable (unmasked, uppercase) sequence length:

$$occupancy\,\% = (covered\ non - N\ positions\ /\ total\ non - N\ positions)\ \times 100$$

This correction ensures that occupancy values reflect genuine sequence coverage rather than assembly completeness and is particularly important for Y-linked sequences where assemblies are often fragmented and gap-rich.

S3.3.11. *Command-line parameters*

The pipeline is executed via command line and accepts the following parameters, which control the stringency and scope of the analysis:

`--fasta_file`: Input FASTA file containing the query sequences to be classified.
`--strict_identity`: Restricts the identity threshold search to a narrower range (98.0–100.0%) instead of the default range (95.0–100.0%). Recommended for single-copy genes with high-quality reference sequences.
`--hit_weight_factor`: Controls the penalty applied to excessive BLAST hits when selecting the optimal identity threshold (default: 2.0). Setting this to 0 removes the hit penalty entirely.
`--Y_linked`: Enables (default) or disables filtering of BLAST hits to retain only those mapping to YGS-validated Y-linked contigs. Accepts 'yes' or 'no'.
`--assembly_aware`: Enables the assembly-aware classification module, which distinguishes between genuine pseudogene signals and assembly fragmentation artifacts. Recommended for multi-copy gene families and sequences known to be poorly assembled.
`--permissive_thresholds`: Lowers the presence/absence thresholds from 80%/10% to 70%/15% for all sequences. Useful for repetitive and multi-copy loci where intermediate occupancy values are expected due to the inherent difficulty of assembling such regions.

`--scaffolds`: Enables scaffold-aware mode, which relaxes pre-filtering rules and adapts occupancy thresholds to sequence length. Intended for classifying long genomic scaffolds that may span multiple fertility regions.

`--YGS`: Path to a YGS classification file containing P_VSC_UK values, which the pipeline uses to refine pre-filtering decisions (distinguishing between true Y-linked sequences with assembly issues and autosomal contamination).

`--read_validation`: Enables read-based validation using minimap2, which independently classifies sequences based on Illumina read coverage and integrates the results with BLAST-based classification. Recommended for all standard analyses; can be disabled to reduce runtime for large input sets when assembly-based classification alone is sufficient.

`--threads`: Number of threads for BLAST and minimap2 (default: 4).

`--blast_db_dir`: Directory containing the BLAST databases (default: current directory).

`--read_db_dir`: Directory containing paired-end read files for minimap2 validation (default: minimap2_read_databases).

`--y_linked_gi_dir`: Directory containing Y-linked contig identifier lists for each strain (default: current directory).

`--output`: Prefix for all output files. If provided, a directory is created and all output files (BLAST results, BAM files, log file, and classification summary) are written to it.

`--resume`: Skips BLAST and/or minimap2 alignment steps if output files from a previous run are detected, allowing the pipeline to be re-run with different classification parameters without repeating the computationally expensive alignment steps.

`--trust_read_pattern`: When enabled, accepts read-based regional assignments in cases where the BLAST branch returns Undetermined. The resulting classifications are capped at a maximum confidence of 35 and annotated as "*read-pattern only*" in the output. Requires `--read_validation`.

The pipeline produces a tab-delimited output file (kennison_occupancy_out.txt) containing, for each query sequence: occupancy percentages and hit counts across all eight databases, P_VSC_UK values (if provided), the inferred chromosomal location, confidence score and level, selected identity threshold, and, when read validation is enabled, the read-based classification, support score, and

integrated final assignment. A detailed log file records all classification decisions and intermediate values.

S3.4. Cross-assembly localization of FlyBase-annotated *Su(Ste)* sequences

To verify the genomic position of three FlyBase-annotated *Su(Ste)* transcripts (*CR42424, CR42429, CR42431*) that the pipeline assigned to kl-2 despite their kl-1/centromere FlyBase coordinates, each transcript was BLASTed (blastn, e-value cutoff 1×10^{-4}) against the *D. melanogaster* Release 6 (R6) assembly and the Chang and Larracuente (2019) PacBio (CL) assembly, alongside the canonical *Su(Ste)* transcript *CR40820* and the *WDY* gene as positional landmarks. Best-hit coordinates are reported in *Supplementary Table S8*. Equivalent searches were also performed against the LILAP PacBio HiFi and ONT Nanopore assemblies; results from these long-read assemblies are not tabulated (see *Supplementary Results* for interpretation).

Table S8. R6 and CL coordinates of three FlyBase-annotated *Su(Ste)* sequences and positional landmarks. Best-hit blastn coordinates (subject start–end; ≥99.8% identity over the full transcript length). Landmarks are the canonical *Su(Ste)* transcript *CR40820* and the *WDY* gene.

Sequence	R6 position	CL contig	CL position
Su(Ste):CR40820 (canonical)	953,256–954,313	Y_Contig17	235,721—234,664
WDY	2,411,979–2,413,059	Y_scaffold4	3,076,452—3,077,531
lncRNA:CR40947	348—354 (armU)	Y_scaffold4	2,538,604—2,538,257
CR42431	2,559,809–2,561,503	Y_Contig17	335,886—334,197
CR42424	2,562,676–2,564,365	Y_Contig17	335,884—334,638
CR42429	2,562,678–2,563,924	Y_Contig17	338,739—337,045

S3.5. Application to external Supplementary Datasets

S3.5.1. Classification and Cross-Assembly Validation of Unlocalized R6 Scaffolds

Reference assemblies: The unlocalized scaffolds of the *D. melanogaster* Release 6 (R6) assembly were validated against four independent reference assemblies of the same species, spanning a range of sequencing technologies and assembly approaches:

i. R5 — the *D. melanogaster* Release 5 assembly (Hoskins et al. 2007; Hoskins et al. 2015), the predecessor of R6 and the most directly comparable Sanger-based reference.
ii. CL — the long-read PacBio assembly of Chang and Larracuente (2019), which used PacBio reads to resolve repetitive Y-chromosome regions inaccessible to Sanger sequencing.
iii. LILAP — a PacBio HiFi assembly generated *de novo* from reads reported by Jia et al. (2024) (SRX20676066, SRX20676067) using hifiasm v0.25.0-r726 with default parameters.
iv. ONT — a Nanopore assembly generated from reads reported by Kim et al. (2024)using a Y-enriched read selection strategy. All ONT reads longer than 45 kb were retained. To improve recovery of Y-linked sequences, additional ONT reads between 1 and 45 kb with a YGS score ≥20% were selected and combined with the >45 kb read set. The resulting dataset (417,609 reads) was assembled using Hifiasm v0.25.0 (--ont --primary, -l 1, default filtering parameters). Primary contigs were extracted from the p_ctg.gfa file to generate the final assembly, while alternative contigs were retained separately.

For LILAP and ONT, contaminant sequences were removed with FCS-GX (Astashyn et al. 2024). The four assemblies represent independent reconstructions of the *D. melanogaster* Y chromosome, providing a stringent test of Y-linkage assignments based on the R6 unlocalized scaffolds.

Pipeline classification: We extracted the unlocalized scaffolds from the R6 assembly using *seqtk seq*. This set comprises both truly unplaced sequences and scaffolds assigned to chromosomal regions based on comparative genome hybridization in embryos carrying chromosome deficiencies. Uninformative repetitive sequences were masked using RepeatMasker (version 4.1.8), and the filtered scaffolds were analyzed with the Digital Kennison pipeline under the parameter combination: `--strict`, `--assembly_aware`, `--read_validation`, `--permissive_thresholds`, `--trust_read_pattern`.

Cross-assembly Y-linkage validation: For each comparison, the R6 assembly was aligned as query against the target assembly using minimap2 (`-ax asm5`), and secondary alignments were discarded (`samtools view -F 256`). Primary and supplementary alignments were retained to account for cases

where a single R6 scaffold corresponded to multiple contigs in the target assembly. Scaffold-to-contig correspondence was defined using the target contig recovered in the primary alignment for each R6 scaffold.

The YGS method (Carvalho and Clark 2013) was run independently on each target assembly (k = 24; male and female k-mer count files from Carvalho et al. (2015)), generating a P_VSC_UK value for every contig. For each R6 scaffold, the *P*_VSC_UK value of its corresponding target contig was transferred through the alignment mapping. Scaffolds showing *P*_VSC_UK ≥ 80% across multiple independent assemblies were considered to exhibit robust Y-linkage signal (*Supplementary Dataset S24*).

S3.5.2. Classification of de novo genome assemblies: Windowed composition analysis.

To examine whether unclassified ONT scaffolds were compositionally uniform, we partitioned each scaffold into windows of equal single-copy k-mer content (VSC_K) and computed, per window, the proportion of validated single-copy k-mers that were Y-linked (VSC_UK: male k-mers absent from female reads) versus autosomal or X-linked (VSC_MK: male k-mers present in female reads), following the k-mer framework of YGS (Carvalho and Clark 2013). Windows are defined by k-mer content rather than base-pair length, so window widths in genomic coordinates vary along the scaffold. The number of windows per scaffold was determined adaptively (range: 2–200), with a minimum of 50 VSC_K per window for scaffolds with ≥500 total VSC_K, or 25 VSC_K per window for smaller scaffolds. Scaffolds with fewer than 100 total VSC_K were excluded from the analysis.

Each window was classified by its P_VSC_UK value into one of five categories: strongly Y-linked (≥80%), weakly Y-linked (60–80%), ambiguous (40–60%), weakly autosomal (20–40%), or strongly autosomal (<20%). A scaffold of uniform chromosomal origin produces a flat profile — consistently Y-linked or autosomal along its length — whereas a misjoined scaffold shows a spatial transition between Y-linked and autosomal signals along its length (*Supplementary Figure S4*). This analysis was used as an exploratory diagnostic to characterize scaffolds returned as *Undetermined* by Digital Kennison and was not incorporated into the pipeline's classification itself. The windowed analysis script is available in the project repository (*script1*, see Data Availability,).

S3.5.3. Application to recently transferred Y-linked sequences (Tobler et al. 2017)

Lift from R5 to R6

Tobler et al. (2017) reported a set of consensus Y-linked sequence transfers in *D. melanogaster* using R5 coordinates (Table S9). To classify these transfers with our R6-based pipeline, we extracted query sequences from R5 using samtools (*faidx*) (one extraction per Tobler interval, using the R5 coordinates listed in Table S9) and aligned them to the *D. melanogaster* Release 6 (R6) assembly with blastn (*BLAST*+; E ≤ 1×10^{-4}; no low-complexity filtering; up to 10,000 hits per query).

Table S9. Tobler et al. (2017) consensus Y-linked sequence transfers ('*GeTYs*', Gene Transfer to Y events) in *D. melanogaster*. Coordinates are reported in the R5 assembly as published. The Donor column indicates the autosomal or X-linked source gene(s) when annotated. These consensus transfers were lifted to R6 coordinates and classified with the Digital Kennison pipeline (see methods above)

Consensus	Length	Donor	Tstart	Tend
Dmel_2L_4.46	397	Gs1l,RpL27A	4456606	4457002
Dmel_2L_12.86	711	NA	12858898	12859608
Dmel_2L_19.94	3289	sick	19936154	19939442
Dmel_2L_22.75	510	NA	22752196	22752705
Dmel_2R_0.09	908	NA	99735	100642
Dmel_2R_0.57	1733	NA	566974	568706
Dmel_2R_0.57	1654	NA	737727	739380
Dmel_2R_2.32	1221	NA	2324493	2325713
Dmel_2R_2.32	434	NA	2350137	2350570
Dmel_3L_23.41	563	NA	23412979	23413541
Dmel_3L_24.3	967	NA	24301186	24302152
Dmel_3L_24.3	9969	NA	24432130	24442098
Dmel_3L_24.3	788	NA	24506237	24507024
Dmel_3L_24.3	20643	NA	24503593	24524235
Dmel_3R_17.04	275	CR43975	17042662	17042936
Dmel_3R_20.95	2892	*vig2*	*20948743*	*20951634*
Dmel_3R_20.95	2579	*Bili*	*20956906*	*20959484*
Dmel_3R_20.95	19256	*Clbn*	*20955345*	*20974600*
Dmel_3R_20.95	1573	*Mocs2-CG42503*	*20951654*	*20953226*
Dmel_X_12.7	1150	ade5,CG12717	12656236	12657385
Dmel_X_21.83	3394	NA	21824921	21828314

Consensus: Name of Y-linked transfer after concatenation with all nearby incipient transfers inferred to be from the same transfer event

Tstart: Alignment start position in target (R5 assembly)

Tend: Alignment end position in target (R5 assembly)

Donor = NA: no annotated donor gene

Chaining and Y-linkage filtering

BLAST hits were restricted to R6 scaffolds exhibiting strong Y-linkage signal (P_VSC_UK ≥ 80%; YGS analysis as described above). Because each consensus transfer typically aligned to multiple R6 scaffolds and to multiple HSPs within each scaffold, the retained hits were chained into contiguous aligned intervals using a custom script (*Script S3*), requiring ≥80% identity, ≥200 bp aligned length, ≥15% query coverage, and a maximum inter-HSP gap of 5 kb. Hits that failed to satisfy these criteria were excluded from subsequent analyses.

Special handling for the FDY region

For donors associated with the *FDY* region (*vig2*, *Bili*, *Clbn*, and *Mocs2–CG42503*), BLAST hits were frequently fragmented into short intervals corresponding to dispersed Y-linked pseudogene copies that arose from the original ~11 kb duplication and have since accumulated transposable element insertions and deletions (Carvalho et al. 2015). To retain these biologically relevant matches, chaining thresholds were relaxed (minimum coverage 5%; minimum length 100 bp).

The resulting chained intervals were converted to genomic coordinates and the corresponding R6 sequences were extracted with `bedtools getfasta`. These extracted sequences served as input to the Digital Kennison pipeline.

Donor-region aggregation

Each consensus transfer typically aligned to multiple chained intervals on the R6 Y chromosome, each receiving its own per-interval classification from the Digital Kennison pipeline. To produce a single donor-level classification suitable for biological interpretation, per-interval classifications were aggregated using a custom script (*Script S4*).

For each donor, we computed: the number of Y-linked hits (*n_hits*), the modal inferred location (*final_modal*), the fraction of hits supporting the modal assignment (*final_modal_frac*), the number of

distinct non-*Undetermined* locations (*final_n_distinct*), the mean confidence score, and the BLAST–read agreement fraction.

Donors were assigned to one of four categories based on these metrics:

i. Resolved: a single hit with a definitive classification, or ≥2 hits with *final_modal_frac* ≥ 0.8 and a single dominant location.

ii. Multicopy single region: ≥3 hits with a consistent dominant location (final_modal ≠ Undetermined; final_modal_frac ≥ 0.6) and either mean confidence ≥70 or BLAST–read agreement ≥0.7. Cases meeting only the modal-fraction criterion (0.6 ≤ final_modal_frac < 0.7) without supporting confidence or read-agreement evidence were reclassified as Dispersed.

iii. Dispersed: ≥3 hits that did not meet the criteria for Resolved or Multicopy single region, including donors with no dominant location (final_modal_frac < 0.6) as well as those with moderate modal support (0.6 ≤ final_modal_frac < 0.8) that lacked sufficient confidence (final_mean_conf < 70) and BLAST–read agreement (<0.7). These patterns are consistent with repeat- or transposable element–derived sequences distributed across multiple Y regions.

iv. Insufficient evidence: cases in which the pipeline did not produce a reliable classification (final_modal = Undetermined).

For the *FDY* region (*vig2*, *Bili*, *Clbn*, and *Mocs2–CG42503*; consensus transfer Dmel_3R_20.95), individual donor entries were collapsed into a single consensus-transfer unit at the aggregation step, since these sequences derive from a single ~11 kb duplication event (Carvalho et al. 2015) and were already analyzed with relaxed chaining thresholds (above).

S3.6. Auxiliary scripts

The following scripts, distributed with the pipeline in the project GitHub repository (see Data availability), were used to generate the analyses reported here.

- **Script S1** - `prefilter_reads_remove_autosomal.py`: Pre-filter Illumina reads to remove autosomal contamination prior to minimap2 validation. Used in: S3.3.9.
- **Script S2**– `plot_ygs_windows.py` – computes and plots windowed Y vs autosomal k-mer composition along user-specified scaffolds. Used in: S3.5.2, Fig. S4.
- **Script S3** – `chain_blast_hsps.py`: merges co-linear, same-strand BLAST HSPs fragmented by internal divergence, indels, or repetitive content. Used in S3.5.3.

- **Script S4** - `aggregate_donors.py`: aggregates Digital Kennison classifications per donor region for the Tobler et al. (2017) transfer set. Used in: S3.5.3, Table S9.
- **Script S5** - `plot_classification_confidence.py`*:* generates a two-panel figure showing the distribution of confidence scores for each category and the frequency of classification outcomes. Used in: S4.3., Fig. S5.
- **Script S6** - `maskstats.py` - compares repeat (masked) content and length distributions between masked FASTA files; used to test whether Undetermined scaffolds are the repeat-heavy or short ones. *Used in*: S4.5.
- **Script S7** - `read_identity.py` — computes per-scaffold read-mapping identity from the Kennison per-database BAMs; used to test whether ONT scaffolds carry lower read identity than PacBio. *Used in*: S4.5.
- **Script S8** - `merge_cross_assembly_ygs.py`: merges per-target-assembly YGS mapping files into a single wide table keyed by R6 scaffold. *Used in*: S3.5.1; reproduces Supplementary Dataset 2, sheet S24.
- **Script S9** – `YGS_plot.py`: Dedicated plotting script for YGS output files. Used in *Fig S2*.

S4. Supplementary Results and Discussion

S4.1. Evaluation of Classification Accuracy

S4.1.1. *Single-Copy Gene Set*

For the single-copy Y-linked gene set, we evaluated seven parameter combinations (Table S5), keeping the hit-weight factor fixed at 2.0 (default). Chromosomal assignments were consistent across all runs for 12 of the 13 genes (Table S10). The exception was *FDY*, whose ~98% identity to the autosomal paralog vig2 resulted in intermediate BLAST occupancy (~50%) across most databases, masking the true presence/absence signal. Without read validation, *FDY* was either left unassigned or incorrectly placed in the kl-1 region with a very low confidence score (33.7) due to an artifactual absence in the kl-1^{-} assembly (Runs 2 and 3). Correct classification to the centromeric region was achieved only with *--read_validation* enabled (Run 7). Detailed results for all runs are available in *Supplementary Dataset 2* (*sheets S1–S7*).

The pipeline is based on the expectation that a gene absent from a given translocation strain will show low occupancy in that strain's database and high occupancy in all others. This assumption can break down in the BLAST-based analysis when a gene has a highly similar autosomal paralog, as cross-mapping inflates occupancy across all databases regardless of the gene's true location. *FDY* was the only gene whose classification varied across runs (Table S10): without read validation, it was either left unassigned or incorrectly placed in kl-1 with low confidence (33.7), whereas correct centromeric localization required the `--read_validation` option (Run 7).

Table S10. Summary of classification accuracy of known Y-linked single-copy genes relative to known chromosomal locations across test runs.

Genes	Run 1	Run 2	Run 3	Run 4	Run 5	Run 6	Run 7	Notes
kl-2	✓	✓	✓	✓	✓	✓	✓	1
kl-3	✓	✓	✓	✓	✓	✓	✓	1
WDY	✓	✓	✓	✓	✓	✓	✓	1
ORY	✓	✓	✓	✓	✓	✓	✓	1
Ppr-Y	✓	✓	✓	✓	✓	✓	✓	1
kl-5	✓	✓	✓	✓	✓	✓	✓	1
ARY	✓	✓	✓	✓	✓	✓	✓	1
CCY	✓	✓	✓	✓	✓	✓	✓	1
PRY	✓	✓	✓	✓	✓	✓	✓	1
CG41561	✓	✓	✓	✓	✓	✓	✓	1
Pp1-Y1	✓	✓	✓	✓	✓	✓	✓	1
Pp1-Y2	✓	✓	✓	✓	✓	✓	✓	1
FDY	Und	kl-1	kl-1	Und	Und	Und	✓	2

1 = Standard threshold success

2 = Resolved with --read_validation

S4.1.2. *Mst77Y Gene Cluster*

Under baseline settings (Run 8), all 18 copies were correctly assigned to the centromeric region. Enabling strict identity thresholds (Run 9) caused three pseudogenes (*Mst77Y-8*, *Mst77Y-11ψ*, and *Mst77Y-18ψ*) to become unclassified, as the higher alignment stringency discarded divergent but legitimate hits in poorly assembled copies. Adding `--assembly_aware` mode (Run 10) recovered all three by reinterpreting their intermediate occupancy values as fragmentation artifacts rather than genuine absence, restoring 100% accuracy. All subsequent configurations (Runs 11–14) maintained full accuracy (Table S11).

Classification paths varied with biological status in a predictable way. Intact, well-assembled copies were classified through the standard centromere pattern — high occupancy across all six fertility databases and low occupancy in V8 and V24. A subset of pseudogenized copies required non-standard classification paths: *Mst77Y-3ψ* and *Mst77Y-5ψ* required relaxed uniform centromere detection, in which consistently intermediate occupancy across databases was interpreted as fragmented rather than absent signal; Mst77Y-11ψ required assembly artifact resolution, in which intermediate values were promoted to presence before pattern matching (Table S12).

Read-based validation (Run 14) confirmed all 18 classifications through the standard centromere path, including all pseudogenes. Detailed results for all runs can be found in *Supplementary Dataset 2* (*sheets S8–S14*).

Table S11. Summary of classification accuracy for the Mst77Y gene family relative to known chromosomal locations across test runs

		Correct		Undetermined		Incorrect	
Runs	Parameters added	N	%	N	%	N	%
Run 8	Baseline	18	100%	0	0%	0	0%
Run 9	+ strict identity	15	83.3%*	3	16.7%	0	0%
Run 10	+ assembly aware	18	100%	0	0%	0	0%
Run 11	+ Y-linked filter	18	100%	0	0%	0	0%
Run 12	+ relaxed thresholds	18	100%	0	0%	0	0%
Run 13	+ scaffold mode	18	100%	0	0%	0	0%
Run 14	+ read validation	18	100%	0	0%	0	0%

* Sequences classified as “undetermined (possible pseudogene)” due to ambiguous or incomplete occupancy patterns: Mst77Y-8, Mst77Y-11 ψ, Mst77Y-18 ψ

Table S12. Digital Kennison classification paths under the optimal configuration

Genes	Blast path	Read path
Mst77Y-1	Std	Std
Mst77Y-2	Std	Std
Mst77Y-3 ψ	Rlx-U	Std
Mst77Y-4	Std	Std
Mst77Y-5 ψ	Rlx-U	Std
Mst77Y-6 ψ	Std	Std
Mst77Y-7	Std	Std
Mst77Y-8	Std	Std
Mst77Y-9	Std	Std
Mst77Y-10 ψ	Std	Std
Mst77Y-11 ψ	Art	Std
Mst77Y-12	Std	Std
Mst77Y-13	Std	Std
Mst77Y-14 ψ	Std	Std
Mst77Y-15 ψ	Std	Std
Mst77Y-16 ψ	Std	Std
Mst77Y-17 ψ	Std	Std
Mst77Y-18 ψ	Std	Std

Std = Standard centromere – All 6 fertility databases high, V8/V24 low

Rlx = Relaxed centromere (≥5 high) – 5/6 fertility databases high, none low

Rlx-U = Relaxed centromere (uniform) = ≥5 databases intermediate but consistent

Art = Assembly artifact resolved – Intermediate values promoted, then pattern matched

S4.1.3. *Y-Linked Non-Coding RNA Set*

Classification accuracy across all eight parameter configurations is summarized in *Tables S13* and *S14*; complete run-by-run results are provided in *Supplementary Dataset 2* (*sheets S15–S22*).

The read-based approach, which performed strongly for single-copy genes and for the *Mst77Y* cluster, was uninformative for this dataset. Rather than producing the expected pattern of low coverage in the relevant translocation-deficient dataset and high coverage elsewhere, read-based profiles showed near-uniform high coverage across all six databases, masking the regional signal. Read-based classification therefore reduced rather than improved accuracy under most parameter configurations (Supplementary *Tables S13* and *S14*; *Supplementary Dataset 2, sheets S15–S22*). This behavior reflects a fundamental limitation of read-based mapping for high-copy paralog families: short reads derived from any *Su(Ste)*

locus align with high identity to other *Su(Ste)* copies, causing the coverage profile of a query sequence to reflect total family abundance rather than the presence or absence of a specific genomic copy.

BLAST-based classification, by contrast, retained discriminatory power once the hit-weight factor was elevated from the default of 2.0 to 5.0. This adjustment down-weights the redundant cross-mapping alignments characteristic of multi-copy families, recovering the regional signal otherwise masked by spurious hits (Run 22; *Tables S13* and *S14*).

Within the canonical *Su(Ste)* cluster, parameter-sensitive misclassifications under suboptimal configurations were non-randomly directed: in 15 of 18 events across Runs 15–21, misclassified Su(Ste) sequences were assigned to the centromere region rather than to kl-2 (the remaining three events produced ambiguous kl-3/kl-2 classifications). Six canonical *Su(Ste)* sequences (*CR42420*, *CR42421*, *CR42426*, *CR42427*, *CR42438*, *CR45794*) were affected. Under Run 22, all but one (*CR42427*, ambiguous) were correctly classified to kl-2. The *PCKR* sequence *CR40947* was correctly mapped to the kl-1/centromere region under all tested configurations, including those in which canonical *Su(Ste)* sequences were misclassified (Table S13).

Table S13. Digital Kennison classification accuracy for Y-linked non-coding RNA sequences across parameter settings.

Gene	Run 15	Run 16	Run 17	Run 18	Run 19	Run 20	Run 21	Run 22
CR40629-RC	×	×	×	×	×	×	×	×
Su(Ste):CR40820	✓	✓	✓	✓	✓	✓	✓	✓
Su(Ste):CR41533	✓	✓	✓	✓	✓	✓	✓	✓
Su(Ste):CR42407	✓	✓	✓	✓	✓	✓	✓	✓
Su(Ste):CR42410	✓	✓	✓	✓	✓	✓	✓	✓
Su(Ste):CR42412	✓	✓	✓	✓	✓	✓	✓	✓
Su(Ste):CR42414	✓	✓	✓	✓	✓	✓	✓	✓
Su(Ste):CR42415	✓	✓	✓	✓	✓	✓	✓	✓
Su(Ste):CR42416	✓	✓	✓	✓	✓	✓	✓	✓
Su(Ste):CR42417	✓	✓	✓	✓	✓	✓	✓	✓
Su(Ste):CR42419	✓	✓	✓	✓	✓	✓	✓	✓
Su(Ste):CR42420	×	×	×	✓	✓	✓	✓	✓
Su(Ste):CR42421	✓	✓	✓	✓	×	×	×	✓
Su(Ste):CR42422	✓	✓	✓	✓	✓	✓	✓	✓
Su(Ste):CR42425	✓	✓	✓	✓	✓	✓	✓	✓
Su(Ste):CR42426	×	×	×	✓	✓	✓	✓	✓
Su(Ste):CR42427	×	×	×	×	×	×	×	Amb
Su(Ste):CR42428	✓	✓	✓	✓	✓	✓	✓	✓
Su(Ste):CR42430	✓	✓	✓	✓	✓	✓	✓	✓
Su(Ste):CR42432	✓	✓	✓	✓	✓	✓	✓	✓
Su(Ste):CR42438	✓	✓	✓	×	✓	✓	✓	✓
Su(Ste):CR42444	✓	✓	✓	✓	✓	✓	✓	✓
Su(Ste):CR45794	✓	✓	✓	×	✓	✓	✓	✓
Su(Ste):CR45795	✓	✓	✓	✓	✓	✓	✓	✓
Su(Ste):CR45796	✓	✓	✓	✓	✓	✓	✓	✓
Su(Ste):CR42424	×	×	×	×	×	×	×	×
Su(Ste):CR42429	×	×	×	×	×	×	×	×
Su(Ste):CR42431	×	×	×	×	×	×	×	×
lncRNA:CR40947-RB	✓	✓	✓	✓	✓	✓	✓	✓

✓ = classification matching the known chromosomal location

× = incorrect classification

Amb = ambiguous classification (kl-2/centromere ambiguity)

Table S14. Summary of classification accuracy for Y-linked non-coding RNA sequences across test runs

Run	Parameters added	Correct	Undetermined	Incorrect
15	Baseline	22 (75.9%)	0	7 (24.1%)
16	+ strict identity	22 (75.9%)	0	7 (24.1%)
17	+ assembly aware	22 (75.9%)	0	7 (24.1%)
18	+ Y-linked filter	22 (75.9%)	0	7 (24.1%)
19	+ relaxed thresholds	23 (79.3%)	0	6 (20.7%)
20	+ scaffold mode	23 (79.3%)	0	6 (20.7%)
21	+ read validation	23 (79.3%)	0	6 (20.7%)
22	Hit weight = 5	24 (82.7%)	1 (3.4%)	4 (13.8%)

S4.1.4. *Cross-assembly evidence for kl-1/centromere placement of three FlyBase-annotated Su(Ste) sequences*

In R6, where the Y chromosome is represented as a single contiguous assembly, the three transcripts cluster within a ~5-kb window at 2,559,809–2,564,365, located ~150 kb distal to *WDY* and ~1.6 Mb downstream of the canonical *Su(Ste)* locus (*Supplementary Table S8*). This places them in the kl-1/centromere region rather than within the main *Su(Ste)* cluster at kl-2. The same arrangement is recovered in the CL assembly: the three transcripts share a single contig, distinct from the contig bearing *WDY*. Both assemblies thus place the three transcripts in the genomic region assigned to the *PCKR* (*pseudo-CK2β* repeat) cluster by Chang and Larracuente (2019), located at bands h14–h16.

Equivalent searches against the LILAP PacBio HiFi and ONT Nanopore assemblies recovered the three transcripts but distributed them across multiple contigs in patterns that differed between the two assemblies, with one transcript (*CR42431*) clustering with canonical *Su(Ste*) in both assemblies. This is consistent with the well-documented difficulty of resolving paralogous repeat clusters in long-read-only assemblies of the *D. melanogaster* Y chromosome, where sequence similarity between *Su(Ste)* and *PCKR* copies — including documented chimeric variants generated by gene conversion (Chang and Larracuente, 2019, Figure 4C) — can lead assemblers to co-locate sequences that are physically distant in the reference assembly.

The pipeline's regional assignment of these three transcripts to the kl-2 region, despite their kl-1/centromere position, reflects the abundance asymmetry between the two paralogous clusters: cross-mapping reads from the much larger *Su(Ste)* cluster at kl-2 dominate the occupancy signal of the smaller post-*WDY PCKR* cluster, pulling the regional assignment toward kl-2 even after hit-weight factor adjustment (see main text).

S4.1.5. *Cross assembly analysis of CR40629-RC*

To investigate the genomic context of *CR40629-RC* independently of the R6 reference assembly, we performed a series of targeted BLAST+ BLASTN searches across multiple *D. melanogaster* Y chromosome assemblies generated using different sequencing technologies and assembly strategies.

We first queried the heterochromatin-enriched PacBio assembly from Chang and Larracuente (2019). This search returned multiple hits to *CR40629-RC* at 82–87% identity, all on Y_scaffold7 — the scaffold that also contains the kl-2 region genes *kl-2* and *Ppr-Y*, annotated as immediate neighbors of *CR40629-RC* in FlyBase (release 6.55). No high-identity match (>95%) to the full transcript was found, indicating that the locus is incompletely assembled or misrepresented in this dataset. BLAST+ blastn analysis of the FlyBase R6 assembly, by contrast, returned a single perfect match (Y:1,122,988–1,123,872; 100% identity, 100% coverage), corresponding to the annotated *CR40629-RC* locus, with additional ~85% identity hits scattered across Y-linked scaffolds representing paralogous or pseudogenized copies. The ONT assembly (HiFiasm; Nanopore Q20+ reads from Kim et al. (2024)) likewise recovered the full-length transcript (100% identity) but also assembled more than 50 related sequences (~85% identity) on a separate Y-linked contig, preventing precise localization of the individual paralogs.

To test whether the paralogous copies inferred from R6 are genuinely co-located with *CR40629-RC*, as their clustered R6 positions suggest, we extracted all sequences ≥400 bp and ≥80% identical to *CR40629-RC* from the R6 assembly (56 sequences, including the canonical transcript) and classified them under the optimized configuration (`--Y_linked, --assembly_aware, --strict_search, --read_validation, --hit_weight 5`). The classifications were not uniform (Table S15; *Supplementary Dataset 2, sheet S23*): of the 55 paralogs, 52 were assigned to the kl-2 region (confidence 26.9–98.2, median 41.65), two to the ks-2 region at low confidence (28.1), and one was ambiguous (centromere vs. kl-2). *CR40629-RC* was the only sequence assigned to kl-5 (confidence 100). It is therefore distinguished from its paralogs by its occupancy pattern across the deficiency panel rather than by sequence similarity.

Specifically, two non-mutually exclusive scenarios are consistent with the data: (i) a misassembly at the *CR40629-RC* locus in R6, placing an otherwise kl-5 sequence within the kl-2 region paralog cluster, or (ii) an annotation inherited from an earlier, more fragmented assembly that was not subsequently revised. Distinguishing between these scenarios is beyond the scope of this study. Nonetheless, the result illustrates a broader point: an independent computational mapping framework can identify annotations

that conflict with underlying sequence evidence, even when those annotations have remained unchallenged in a widely used reference.

Table S15. Digital Kennison classification of the *CR40629* paralog family. Approximate positions refer to coordinates in the *D. melanogaster* Release 6 (R6) assembly. ψ denotes putative pseudogenes based on sequence divergence from the annotated *CR40629-RC* transcript. Confidence scores reflect the strength of evidence supporting each classification.

	scaffold	approximate position	Pipeline assignment	Confidence score
CR40629-RC	Y	1122988-1123872	kl-5	100
CR40629-like 1 ψ	Y	1006221-1006644	kl-2	73.4
CR40629-like 2 ψ	Y	866244-866651	ks-2	28.1
CR40629-like 3 ψ	Y	872110-872521	kl-2	67.2
CR40629-like 4 ψ	Y	896870-897284	kl-2	75.3
CR40629-like 5 ψ	Y	2518434-2518848	kl-2	75.3
CR40629-like 6 ψ	Y	2532786-2533200	kl-2	50.2
CR40629-like 7 ψ	Y	2538521-2538935	kl-2	75.3
CR40629-like 8 ψ	Y	2541390-2541804	kl-2	75.3
CR40629-like 9 ψ	Y	2544259-2544673	kl-2	75.3
CR40629-like 10 ψ	Y	2546786-2547200	kl-2	75.3
CR40629-like 11 ψ	Y	2563263-2563677	kl-2	75.3
CR40629-like 12 ψ	Y	2594209-2594623	kl-2	75.3
CR40629-like 13 ψ	Y	2529953-2530367	kl-2	26.3
CR40629-like 14 ψ	Y	2549314-2549728	kl-2	39.6
CR40629-like 15 ψ	Y	2552183-2552597	kl-2	39.6
CR40629-like 16 ψ	Y	2555052-2555466	kl-2	39.6
CR40629-like 17 ψ	Y	2557895-2558309	kl-2	40.1
CR40629-like 18 ψ	Y	2566102-2566516	kl-2	27.3
CR40629-like 19 ψ	Y	2568941-2569355	kl-2	27.3
CR40629-like 20 ψ	Y	2571780-2572194	kl-2	27.3
CR40629-like 21 ψ	Y	2574614-2575028	kl-2	42.6
CR40629-like 22 ψ	Y	2577456-2577870	kl-2	27.3
CR40629-like 23 ψ	Y	2580296-2580710	kl-2	27.3
CR40629-like 24 ψ	Y	2583128-2583542	kl-2	27.3
CR40629-like 25 ψ	Y	2588841-2589255	kl-2	40.1
CR40629-like 26 ψ	Y	914988-915413	kl-2	52.7
CR40629-like 27 ψ	Y	945389-945805	kl-2	55.5
CR40629-like 28 ψ	Y	2512742-2513139	kl-2	45.3
CR40629-like 29 ψ	Y	2535659-2536072	kl-2	49.9
CR40629-like 30 ψ	Y	2560393-2560807	kl-2	26.9
CR40629-like 31 ψ	Y	2585989-2586403	kl-2	26.9

CR40629-like 32 ψ	Y	2591339-2591753	kl-2	26.9
CR40629-like 33 ψ	Y	2597048-2597462	kl-2	27.3
CR40629-like 34 ψ	Y	931121-931546	Centromere/ kl-2	60
CR40629-like 35 ψ	Y	909820-910228	kl-2	77.4
CR40629-like 36 ψ	Y	903502-903916	kl-2	43.7
CR40629-like 37 ψ	Y	850998-851406	kl-2	40.6
CR40629-like 38 ψ	Y	858097-858505	kl-2	98.2
CR40629-like 39 ψ	Y	908358-908766	kl-2	98.2
CR40629-like 40 ψ	Y	1000304-1000712	kl-2	40.6
CR40629-like 41 ψ	Y	1034396-1034821	kl-2	44.6
CR40629-like 42 ψ	Y	953344-953761	kl-2	38.4
CR40629-like 43 ψ	Y	2659961-2660378	kl-2	38.4
CR40629-like 44 ψ	Y	2665407-2665824	kl-2	38.4
CR40629-like 45 ψ	Y	925739-926463	kl-2	34.5
CR40629-like 46 ψ	Y	920351-920776	kl-2	42.1
CR40629-like 47 ψ	Y	848856-849264	kl-2	63.3
CR40629-like 48 ψ	Y	985062-985477	kl-2	41.2
CR40629-like 49 ψ	Y	934742-935162	kl-2	76.6
CR40629-like 50 ψ	Y	1016829-1017229	kl-2	18.9
CR40629-like 51 ψ	Y	2645777-2646177	kl-2	18.9
CR40629-like 52 ψ	Y	979632-980064	kl-2	27.5
CR40629-like 53 ψ	211000022280494	2716-3139	kl-2	73.4
CR40629-like 54 ψ	211000022278436	2322-2729	ks-2	28.1
CR40629-like 55 ψ	211000022280481	1782-2191	kl-2	68.8

S4.2. Centromere contig classification

We applied Digital Kennison to the five *D. melanogaster* centromere contigs identified by Chang et al. (2019): Contig79 (CenX), tig00057289 (Cen2), 3R_5 (Cen3), Contig119 (Cen4), and Y_Contig26 (CenY). Sequences were obtained from File.S8 of the associated Dryad repository (doi:10.5061/dryad.rb1bt3j) and run unmasked, since the repetitive content of centromeric DNA is the signal of interest.

Under default parameters (hit-weight factor 2.0), none of the five contigs received a regional assignment, including Y_Contig26 (*Supplementary Dataset 2, sheet S28*). For Y_Contig26, the BLAST branch recovered the qualitative centromeric pattern —occupancy across all six fertility databases and near-absence from V8 and V24 (0.1% and 0.7%, respectively) — but absolute occupancy values remained low (11.7–30.6%). This reflects the large number of BLAST hits per database (422–806),

which, under the default hit-weight penalty, drove the per-query identity threshold to 100% and reduced the surviving occupancy below the threshold required for assignment.

Reducing the hit-weight factor to 0 recovered the centromeric assignment for Y_Contig26. Under this same setting, the chromosome 2 centromere contig (tig00057289) also received a Y-linked assignment from the BLAST-branch; however, due to the low confidence of this classification (19.2) and the lack of support from the read-branch, tig00057289 remained *Undetermined*. Intermediate values (0.5–1.0) did not yield a clean assignment for either contig (*Supplementary Dataset 2, sheet S29*).

The three autosomal centromere contigs other than tig00057289 (Contig79, 3R_5, Contig119) showed uniformly low BLAST occupancy across all eight databases (≤2.7%) and did not match any Y-region signature under any parameter setting, returning Undetermined classifications, as expected for non-Y sequences.

The read-based branch did not assign Y_Contig26 to the centromeric region. Although coverage breadth across the six fertility databases was high (88.4–91.7%), consistent with the expected centromeric pattern, the V8 and V24 breadth values (31.2% and 24.2%) exceeded the absence threshold, preventing an incorrect centromeric call. This residual coverage in the female-derived databases reflects reads originating from retroelements that Y_Contig26 shares with autosomal centromeres, principally *G2/Jockey-3*, which is present on all five *D. melanogaster* centromeres and is phylogenetically indistinguishable from genome-wide copies (Chang et al. 2019). Because these elements are genuinely shared between the Y and autosomal centromeres, the cross-mapping signal in V8 and V24 cannot be removed by Y-linkage prefiltering.

S4.3. Assessment of classification confidence

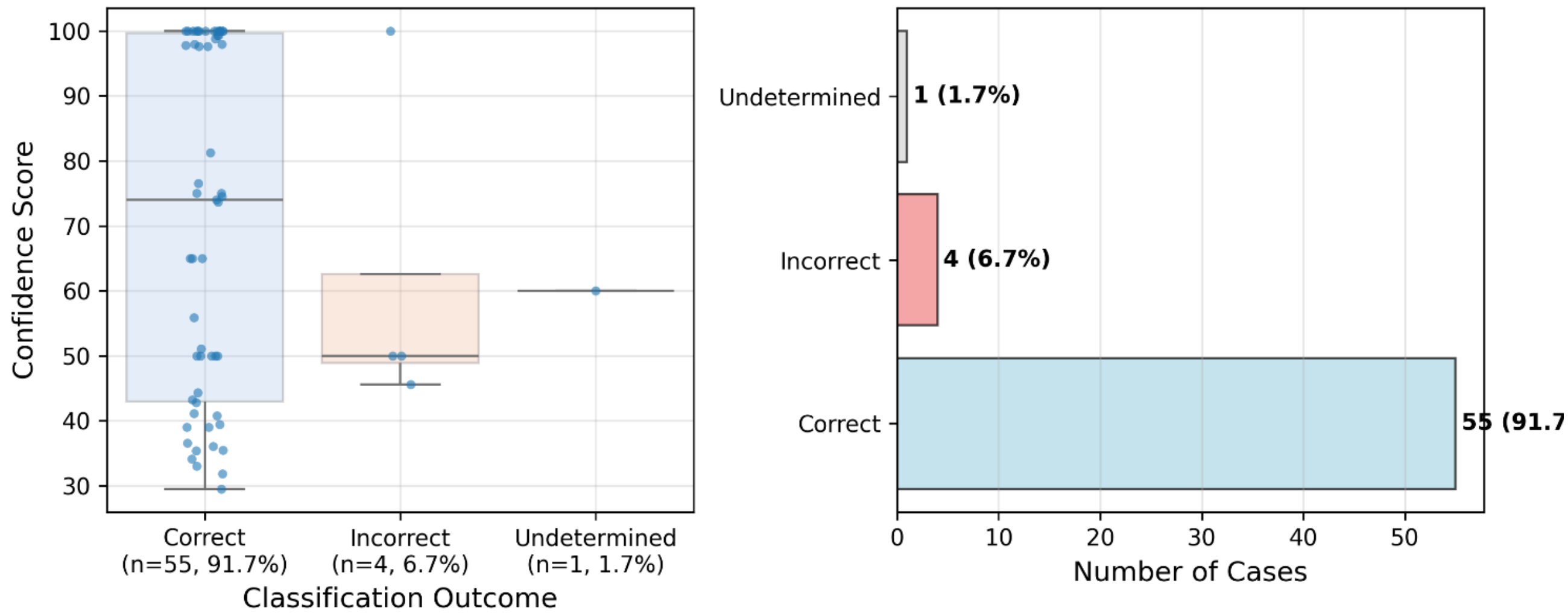

Figure S5. Distribution of confidence scores by classification outcome. The left panel shows confidence score distributions for Correct, Undetermined, and Incorrect classifications. Each point represents an individual sequence, and boxplots summarize the distribution within each category. The right panel shows the number and percentage of sequences in each classification category. Correct classifications span a broad range of confidence scores, reflecting differences in classification difficulty across sequence types: single-copy genes and *Mst77Y* copies typically receive high scores, whereas repetitive *Su(Ste)* sequences often receive lower scores despite being correctly classified. Incorrect classifications (n = 4) cluster at low confidence values, consistent with the scoring system's intended behavior. The sole exception, *CR40629-RC* (confidence score = 100), is discordant with its FlyBase annotation but consistent across all parameter settings, suggesting an annotation inconsistency rather than a classification error (see main text). Figure generated with custom *Script S4* (see Data availability).

Table S16. Classification performance of the Digital Kennison pipeline at successive confidence score thresholds. For each minimum confidence threshold, the table shows the number of sequences evaluated, the number that receive a definitive classification (excluding Undetermined), and three performance metrics: precision (correct/classified), classification rate (classified/total), and overall accuracy (correct/total). Results are based on 60 test sequences analyzed under their respective optimized parameter configurations (Run 7 for single-copy genes, Run 14 for *Mst77Y*, and Run 22 for ncRNAs).

Score threshold	Total	Classified	Correct	Incorrect	Undetermined	Precision	Classification rate	Overall accuracy
≥40	49	48	44	4	1	91.7%	98.0%	89.8%
≥45	44	43	39	4	1	90.7%	97.7%	88.6%
≥50	43	42	39	3	1	92.9%	97.7%	90.7%
≥55	35	34	33	1	1	97.1%	97.1%	94.3%
≥60	34	33	32	1	1	97.0%	97.1%	94.1%
≥65	33	33	32	1	0	97.0%	100.0%	97.0%
≥70	30	30	29	1	0	96.7%	100.0%	96.7%
≥75	27	27	26	1	0	96.3%	100.0%	96.3%
≥80	24	24	23	1	0	95.8%	100.0%	95.8%
≥85	23	23	22	1	0	95.7%	100.0%	95.7%
≥90	23	23	22	1	0	95.7%	100.0%	95.7%
≥95	23	23	22	1	0	95.7%	100.0%	95.7%
100	15	15	14	1	0	93.3%	100.0%	93.3%

S4.4. Cross-assembly evidence for revised chromosomal assignment of five R6 minor scaffolds

The *D. melanogaster* Release 6 (R6) assembly comprises seven chromosome arms (*X, 2L, 2R, 3L, 3R, 4,* and *Y*), the mitochondrial genome, and 1,862 "unlocalized" minor scaffolds. Of these, 884 have been assigned cytologically or genetically to specific chromosomal regions (*X, 2CEN, 3CEN, Y, XY,* and *rRNA*); the remainder carry numeric identifiers and remain unlocalized.

Of the 1,870 scaffolds in the R6 assembly (the 1,862 minor scaffolds plus the eight chromosome-arm and mitochondrial sequences), 904 showed Y-linkage by the YGS method (P_VSC_UK ≥ 80%), excluding the Y chromosome arm itself. Drawn from across both the previously mapped and the unmapped minor scaffolds, these comprise: (i) 12 Y_mapped scaffolds and the XY_mapped scaffold XY_mapped_Scaffold_42_D1648; (ii) 15 Unmapped_Scaffold_* entries; (iii) 871 minor scaffolds with

numeric identifiers, currently unlocalized; and (iv) five scaffolds currently annotated as centromere-proximal on chromosomes 2 or 3:

2Cen_mapped_Scaffold_10_D1684,
2Cen_mapped_Scaffold_43_D1668,
3Cen_mapped_Scaffold_1_D1896_D1895,
3Cen_mapped_Scaffold_31_D1643_D1653_D1791,
3Cen_mapped_Scaffold_41_D1641.

Of the 904 sequences classified as Y-linked, 74.6% were assigned to a specific Y chromosome region by the pipeline (*Supplementary Table S17*; detailed classification results can be found in *Supplementary Dataset 2, sheet S25*). All five exhibit strong, consistent Y-linkage across the four assemblies analyzed (R6, CL, ONT, LILAP; P_VSC_UK ≥ 95.4% in all four; Supplementary Table S18 and Supplementary Dataset 2, sheet S24). In the CL assembly, three were independently annotated as Y-linked; the remaining two (3Cen_mapped_Scaffold_31 and 3Cen_mapped_Scaffold_41_D1641) map to CL contigs with autosomal-style names that nonetheless show P_VSC_UK ≥ 98.3%, making them candidates for misassignment in that assembly as well. Three of the five received specific fertility-region assignments (kl-3, kl-2, and ks-2; *Supplementary Table S18*).

Table S17. Assignment of R6 minor scaffolds to Y chromosome regions and unresolved categories

classification	N	%
Kl-5	39	4.3
Kl-3	75	8.3
Kl-2	115	12.7
Kl-1	229	25.3
Centromere	137	15.2
Ks-1	43	4.8
Ks-2	33	3.7
Amb	1	0.1
Kl-2, kl-1	3	0.3
Und	229	25.3

Amb = ambiguous classification
Und = sequences classified as “undetermined (possible pseudogene)” due to ambiguous or incomplete occupancy patterns

One case warrants specific mention. The R6 scaffold *3Cen_mapped_Scaffold_31_D1643_D1653_D1791* (87.4 kb) and the separate scaffold *3Cen_mapped_Scaffold_41_D1641* correspond to two non-overlapping fragments (_1, 30.5 kb and _8,

71.4 kb) of a larger CL contig group sharing the suffix *D1643_D1653_D1791*. Notably, fragment _8 also matches several R6 unlocalized minor scaffolds, suggesting it likely represents a composite of shorter sequences that remain fragmented in the R6 assembly. Across all matching scaffolds, P_VSC_UK values are consistently high and supported by high mapping quality (*Supplementary Table S19*), indicating strong Y-linkage for the entire contig group. The observed fragmentation in the R6 assembly is therefore consistent with the difficulty of reconstructing such repetitive Y-linked regions from short-read data.

Table S18. Cross-assembly Y-linkage signal and pipeline classification of selected R6 mapped scaffolds. The classification was run under optimized parameters. The confidence score reflects the strength of evidence supporting each classification, ranging from 41 to 71.1. Lower final confidence scores indicate that the relaxed classification path was invoked during the classification process.

R6 assembly contig	% VSC UK				Pipeline assignment	Final confidence score
	R6	CL	ONT	PB (LILAP)		
2Cen_mapped_Scaffold_10_D1684	99.9	96.5	99.7	99.9	kl-3	50
2Cen_mapped_Scaffold_43_D1668	100	98.3	99.9	100	Und	58.5
3Cen_mapped_Scaffold_1_D1896_D1895	99.9	96.5	99.8	99.8	ks-2	67
3Cen_mapped_Scaffold_31_D1643_D1653_D1791	99.9	95.4	99.9	99.9	Und	54.4
3Cen_mapped_Scaffold_41_D1641	100	98.3	99.9	99.9	kl-2	71.1
XY_mapped_Scaffold_42_D1648	100	97.3	99.8	99.9	Und	49.2
Y_mapped_Scaffold_5_D1748_D1610	99.8	96.5	99.8	99.9	kl-1	50
Y_mapped_Scaffold_9_D1573	99.9	97.3	99.9	100	kl-5	50
Y_mapped_Scaffold_12_D1771	99.7	96.5	99.8	99.8	kl-1	50
Y_mapped_Scaffold_15_D1727	99.9	99.6	99.7	99.9	kl-1	50
Y_mapped_Scaffold_18_D1698	99.8	96.5	98.1	99.1	Und	44.7
Y_mapped_Scaffold_20_D1762_D1719	99.2	95.4	99.9	99.9	Und	48.9
Y_mapped_Scaffold_21_D1683_D1693	99.2	82.6	85.2	99.5	centromere	50
Y_mapped_Scaffold_23_D1638	99.7	91.6	86.5	99.4	Und	46.5
Y_mapped_Scaffold_26_D1717	100	24.3	99.2	99.4	Und	41
Y_mapped_Scaffold_30_D1720	98.5	96.5	99.9	99.7	kl-2	50
Y_mapped_Scaffold_34_D1584	100	97.3	99.8	99.9	kl-5	50
Y_mapped_Scaffold_53_D1765	99.9	96.5	99.8	99.8	kl-1	67.3

R6: *D. melanogaster* Release 6 (R6) assembly

CL: Chang and Larracuente 2019 PacBio assembly

ONT: Carvalho et al. 2026 Nanopore assembly generated from reads reported by Kim et al. (2024)

PB(LILAP): LILAP PacBio HiFi assembly generated from reads reported by Jia et al. (2024)

Table S19. Y-linkage signal of R6 scaffolds aligning to Chang and Larracuente (2019) PacBio fragment *D1643_D1653_D1791_8*. Scaffolds were identified by alignment of the CL PacBio assembly against R6 scaffolds using minimap2 (primary alignments only). All alignments received the maximum score of 60, indicating unambiguous placement.

scaffold	Length (bp)	%VSC UK	MAPQ value
3Cen_mapped_Scaffold_41_D1641	22604	100	60
211000022279871	1337	100	60
Unmapped_Scaffold_32_D1773	16157	97.8	60
211000022279659	1103	100	60
211000022280560	4135	100	60
211000022280536	2924	100	60
211000022279770	1280	100	60
211000022280069	1048	100	60

%VSC UK: proportion of valid single-copy k-mers unmatched by female reads (%), with values ≥80% a strong indicative of Y-linkage

MAPQ: minimap2 mapping quality score

S4.5. Classification of unlocalized scaffolds in de novo genome assemblies

Following the procedure described in Methods, candidate Y-linked scaffolds (YGS; P_VSC_UK ≥ 80% and VSC_K > 20) from each assembly were soft-masked with RepeatMasker and classified with read validation and permissive thresholds enabled (LILAP, n = 61; ONT, n = 50), either with or without `--trust_read_pattern`. In both assemblies, enabling `--trust_read_pattern` increased the fraction of scaffolds assigned to specific fertility regions. The effect differed by assembly: in the HiFi assembly, regional assignments rose from 16 to 37 of 61 scaffolds (60.7%), whereas in the ONT assembly, the resolved fraction remained lower, with 17 of 50 scaffolds (34% vs 16% in the standard run) assigned and the majority left Undetermined. (*Supplementary Table S20*; Individual runs: *Supplementary Dataset 2, sheets S30-S35*)

Table S20. Assignment of candidate Y-linked scaffolds from two *de novo* genome assemblies (LILAP-HiFi and ONT) to *D. melanogaster* Y-chromosome fertility regions, including Ambiguous and Undetermined classifications. Digital Kennison was run either without the `-- trust_read_pattern` option (Run 1) or with it (Run 2).

	PB (LILAP)				ONT			
	Run 1		Run 2		Run 1		Run 2	
	N	%	N	%	N	%	N	%
Kl-5	1	1.6	3	4.9	0	0.0	0	0.0
Kl-3	6	9.8	18	29.5	4	8.0	5	10.0
Kl-2	3	4.9	4	6.6	2	4.0	4	8.0
Kl-1	2	3.3	4	6.6	0	0.0	2	4.0
Ks-1	1	1.6	2	3.3	1	2.0	3	6.0
Ks-2	2	3.3	3	4.9	0	0.0	1	2.0
Centromere	1	1.6	2	3.3	0	0.0	1	2.0
ks-1, ks-2	0	0.0	1	1.6	0	0.0	0	0.0
kl-3, kl-2	0	0.0	0	0	1	2.0	1	2.0
undetermined	45	73.8	24	39.3	42	84.0	33	66.0
Total resolved cases (n)	16	26.1	37	60.7	8	16	17	34

The masked fraction was high in both assemblies (means ~97%; medians ≥98%) and did not differ between resolved and unclassified scaffolds in ONT (Mann–Whitney $p = 0.58$). The LILAP (HiFi) assembly carried a marginally higher overall masked fraction than ONT (*Supplementary Table S21*; 90.2% vs 89.5%) yet resolved a greater proportion of scaffolds. Scaffold length did not separate outcomes in the ONT assembly at $\alpha = 0.05$ (medians 191 vs 129 kb; $p = 0.052$). Per-scaffold read-mapping identity was comparable between assemblies (medians of 99.93% vs 99.92% for LILAP and ONT, respectively) and did not differ between resolved and unclassified ONT scaffolds ($p = 0.79$; *Supplementary Table S23*).

Repeating the analysis without repeat-masking did not improve classification. The fraction of resolved scaffolds decreased from 34% to 32.0% in the ONT assembly and from 60.7% to 55.7% in the LILAP assembly. This confirms that repeat content does not provide an informative regional signal at the genome-wide scale and supports repeat masking as the appropriate preprocessing step for scaffold classification (*Supplementary Dataset 2, sheet S32* and *S35*).

Table S21. Repeat content of candidate Y-linked scaffolds in two de novo assemblies (LILAP and ONT). Per-scaffold masked-base fractions and length distributions were computed with *maskstats.py* (Script S6)

Metric	LILAP(HiFi)	ONT
Number of scaffolds	61	50
Total length	15.9Mb	19.35Mb
Repeat-masked bases (%)	90.2	89.5
Interspersed repeats (%)	72.25	66.14
LTR elements (%)	49.92	43.58
Satellites (%)	0.50	2.58

Table S22. Repeat content and scaffold length of de novo assembly candidate scaffolds by classification outcome. Candidate Y-linked scaffolds (P_VSC_UK ≥ 80%, ≥ 20 validated Y-specific single-copy k-mers) from each assembly were classified by Digital Kennison; scaffolds are grouped by whether a fertility region was assigned (resolved) or not (undetermined). Masked fraction and length are per-scaffold medians. *p*-values are Mann–Whitney U (resolved vs. undetermined).

Assembly	Group	n	masked (%) median	masked (%) mean	length (kb) median	length (kb) mean
HiFi (LILAP)	all	61	98.8	97.3	108	261
	resolved	37	98.3	97.1	137	282
	undetermined	24	99.9	97.7	87	229
ONT	all	50	98.0	97.0	146	387
	resolved	17	97.4	96.0	191	623
	undetermined	33	98.7	97.5	129	266

Table S23. Per-scaffold read-mapping identity by assembly and classification outcome. Per-scaffold read-mapping identity was extracted from the Kennison BAMs with *read_identity.py* (Script S7)

Assembly	Group	n	Mean identity (%)	Median identity (%)
HiFi (LILAP)	resolved	37	99.92	99.93
	undetermined	24	99.86	99.89
ONT	resolved	17	99.91	99.92
	undetermined	33	99.91	99.92

Windowed composition analysis

To assess whether unclassified scaffolds were compositionally uniform, we partitioned each scaffold into windows containing equal numbers of single-copy k-mers and, for each window, calculated the proportion of validated single-copy k-mers that were Y-linked (VSC_UK; absent from female reads) or autosomal/X-linked (VSC_MK; present in female reads), following the YGS framework (Carvalho and Clark 2013). Scaffolds of uniform chromosomal origin produce flat profiles that remain consistently Y-linked or autosomal along their entire length, whereas misjoined scaffolds exhibit sharp transitions between the two states. Figure S4 shows these per-window proportions as a function of scaffold position. This analysis was used solely as an exploratory diagnostic and played no role in Digital Kennison classification.

Windowed composition analysis identified a small number of scaffolds with mixed Y-linked and autosomal signals in both the HiFi and ONT assemblies (*Supplementary Table S24*). These scaffolds show sharp transitions between Y-linked and autosomal composition along their length (e.g., ptg000047l, *Supplementary Figure S4 B*), in contrast to scaffolds with uniform Y-linkage signal that were successfully classified (e.g., ptg000005l, *Supplementary Figure S4 A*). Because chimeric scaffolds do not correspond to a single chromosomal location, Digital Kennison appropriately classifies them as Undetermined. Clear misjoins were identified in 3 of 61 LILAP candidate scaffolds (4.9%) and 5 of 50 ONT candidate scaffolds (10.0%); however, excluding chimeric scaffolds from both sets leaves a substantial gap in classification success (LILAP 36/58 = 62.1% vs ONT 17/45 = 37.8%), indicating that misjoins alone do not account for the lower resolution in the ONT data. One predominantly Y-linked chimeric scaffold was successfully classified (ptg000068l → ks-1 in HiFi), indicating that limited chimeric contamination does not necessarily prevent localization when the dominant Y-linked component still produces a diagnostic occupancy pattern.

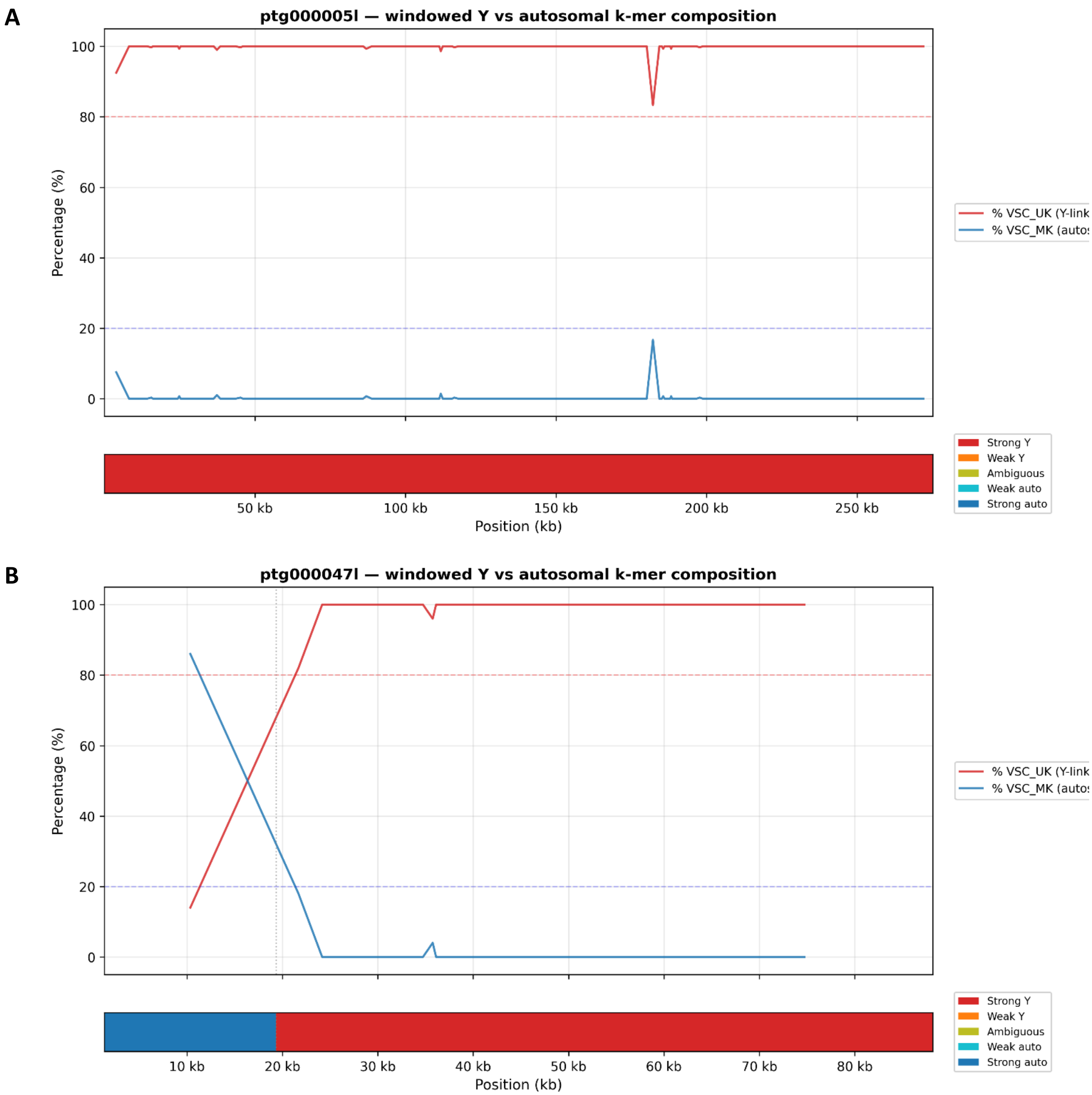

Figure S4. Y-linked versus autosomal k-mer composition for representative ONT scaffolds. For each window, the proportion of validated single-copy k-mers that are Y-linked (VSC_UK; red) or autosomal/X-linked (VSC_MK; blue) is plotted along scaffold position; the lower strip indicates the dominant signal in each window. (**A**) *ptg000005l*, a compositionally uniform Y-linked scaffold, confidently assigned to the kl-3 region. (**B**) *ptg000047l*, which is autosomal across its first ~20 kb and

Y-linked thereafter, was classified as *Undetermined*. Figure generated with the custom Python script *YGS_standalone_forensics.py* (see Data Availability). Windowed P_VSC_UK composition profiles (Fig. S4) were generated with *plot_ygs_windows.py* (Script S2).

Table S24. Scaffolds identified as compositionally mixed by windowed analysis (see *Supplementary Methods*). P_VSC_UK range (min–max) and standard deviation (SD) summarize variation among windows of equal single-copy k-mer content within each scaffold. "Pipeline assignment" indicates the Digital Kennison classification, where applicable. Scaffolds with overall P_VSC_UK below 80% were excluded during YGS pre-screening and were not analyzed (NA). One predominantly Y-linked chimeric scaffold (ptg000068l) nevertheless received a regional assignment, indicating that limited chimeric contamination does not necessarily prevent classification.

Assembly	scaffold	length	P_VSC_UK	P_VSC_UK min(%)	P_VSC_UK max(%)	P_VSC_UK SD	Dominant signal	Pipeline assignment
LIPAP (HiFi)	ptg000026l	87930	97.1	0	100	15.1	strong_Y	Und
	ptg000068l	218235	99.4	0	100	7.4	strong_Y	ks-1
	ptg000095l	283195	98.5	0	100	10.2	strong_Y	Und
ONT	ptg000007l	175119	94.1	0	100	20	strong_Y	Und
	ptg000015l	3887979	86.2	0	100	32.3	strong_Y	Und
	ptg000023l	577253	98.1	0	100	11.7	strong_Y	Und
	ptg000047l	108937	99.3	14	100	7	strong_Y	Und
	ptg000050l	270597	94.5	9.4	100	12.4	strong_Y	Und
	ptg000057l	212971	3	0	82.1	13.1	strong_autosomal	NA

NA = Not analyzed (below YGS threshold)
Und = Undetermined

Table S25. Classification of Y-linked scaffolds in two de novo assemblies (LILAP and ONT) under optimized parameters (`-- trust_read_pattern`). Classification resolution is reported both as the proportion of scaffolds receiving a regional assignment (count-based) and as the proportion of assembled sequence in those scaffolds (length-based). The two metrics can diverge when Undetermined sequence concentrates in a few large scaffolds.

Metric	LILAP(HiFi)	ONT
Number of scaffolds	61	50
Total assembled length	15.9Mb	19.35Mb
Pipeline assignment (n)	37	17(34%)
Classified (bp)	10,448, 543	10,582,707
Resolution by count (%) / by length (%)	60.7% / 65.5%	34% / 54.7%

S4.6. Localizing sequences recently transferred to the Y chromosome – Tobler et al. (2017)

Tobler et al. (2017) reported 21 consensus-transfer entries in *D. melanogaster*. Four of these entries (*vig2*, *Bili*, *Clbn*, and *Mocs2-CG42503*) correspond to a single 11-kb DNA-based duplication characterized by Carvalho et al. (2015) and were therefore aggregated into a single consensus transfer (Dmel_3R_20.95) for our analysis. The remaining multi-row consensus transfers (Dmel_2R_0.57, Dmel_2R_2.32, and Dmel_3L_24.3) reflect Tobler's grouping of nearby incipient transfers rather than independently validated single duplications and were retained as separate input sequences. Two donor regions (2R:94432-100590 and 2R:2324332-2325969) did not pass the Chaining step (see *Supplementary Method: Chaining and Y-linkage filtering*) and were excluded from subsequent analyses.

The FDY locus—comprising *FDY*, *Mocs2-ψ1*, *Clbn-ψ1–ψ4*, *CG42503-ψ1*, and *Bili-ψ1*—corresponds to the *Dmel_3R_20.95* consensus transfer identified by Tobler et al. (2017) and spans a ~55 kb region of the Y chromosome between heterochromatic bands h15 and h20 (Carvalho et al. 2015). To evaluate this locus, we analyzed six input sequences derived from the R6 assembly: three correspond to collapsed HSP chains obtained from BLAST+ blastn searches using *vig2*, *Bili*, and *Clbn* as queries, and three correspond to short (~104 bp) matches to *Mocs2*, representing dispersed pseudogene fragments (*Supplementary Table S26*).

Read-based analysis consistently localized all sequences to the centromeric region, in agreement with the known position of *FDY*. In contrast, BLAST-based classification returned “Undetermined” assignments for the *vig2*, *Bili*, and *Clbn*-derived sequences, as well as for one of the three *Mocs2* fragments (*Supplementary Dataset 2, sheet S27*).

The *Dmel_2R_2.32* transfer (donor *2R:2,350,137–2,350,570*) was classified as a Multicopy_single_region case. Both BLAST- and read-based evidence converged on localization to the *kl-2* region, with moderate support (64% of BLAST hits and 76% of read-based hits assigned to *kl-2*). Agreement between methods was high at the per-hit level (73%), corresponding to 16 of 25 hits sharing the same assignment. The remaining hits were assigned to kl-1, suggesting a predominantly kl-2 signal with limited regional ambiguity.

The Dispersed category comprises consensus transfer regions with genuine classification ambiguity. These cases are typically characterized by multiple hits distributed across several Y-chromosome regions (*kl-1*, *kl-2*, *kl-3*, *kl-5*, *ks-1*, *ks-2*, centromere, and “Undetermined”), with no dominant location. Per-hit confidence scores are generally low or very low, supporting the interpretation that these sequences are derived from transposable elements, satellite DNA, or other repetitive components that are broadly distributed across the Y chromosome.

For example, the *Dmel_2R_0.57* transfer (donor *2R:566,974–568,706*) shows pronounced dispersion, with multiple low-confidence hits scattered across several Y regions and no clear enrichment in any single location. In contrast, the *Dmel_3L_23.41* transfer (donor *3L:23,412,979–23,413,541*), although formally classified as Dispersed, displays a biased distribution of hits: 5 of 9 map to *kl-1* (high confidence), and 3 of 9 to the centromeric region (also high confidence). This pattern suggests a more structured signal, potentially reflecting the presence of this sequence in multiple Y-chromosome regions rather than purely stochastic dispersion (see *Supplementary Dataset 2, sheet S26*, for complete Digital Kennison results).

Table S26. Y-chromosome coordinates of donor-derived sequences associated with the FDY-region transfer. "*Donor*" indicates the autosomal source gene used as query. *Y range* corresponds to the genomic interval spanned by all chained BLAST+ blastn hits for that donor in the *D. melanogaster* Release 6 (R6) assembly. *Y span* denotes the total genomic extent of this interval, while *Total aligned* represents the cumulative length of aligned sequence after HSP chaining.

Donor	Y range	Y span	Total aligned	Notes
vig2	3,021,897–3,024,392	~2.5 kb	~2,400 bp	FDY locus
Mocs2	3,028,326–3,043,033	~14.7 kb	312 bp (3×104)	Three dispersed pseudogene fragments (104 bp each)
Clbn	3,047,733–3,051,262	~3.5 kb	~2,860 bp	Strong multi-HSP chain
Bili	3,049,694–3,051,262	~1.6 kb	~1,567 bp	Multi-HSP chain, fully contained in Clbn region

S5. References

Abad JP et al. 2004. Genomic and cytological analysis of the Y chromosome of *Drosophila melanogaster*: Telomere-derived sequences at internal regions. Chromosoma. 113(6):295–304. https://doi.org/10.1007/s00412-004-0318-0

Astashyn A et al. 2024. Rapid and sensitive detection of genome contamination at scale with FCS-GX. Genome Biol. 25(1). https://doi.org/10.1186/s13059-024-03198-7

Bonaccorsi S, Lohe A. 1991. Fine mapping of satellite DNA sequences along the Y chromosome of *Drosophila melanogaster*: relationships between satellite sequences and fertility factors. Genetics. 129(1):177–189. https://doi.org/10.1093/genetics/129.1.177

Carvalho AB et al. 2015. Birth of a new gene on the Y chromosome of *Drosophila melanogaster*. Proceedings of the National Academy of Sciences. 112(40):12450–12455. https://doi.org/10.1073/pnas.1516543112

Carvalho AB, Clark AG. 2013. Efficient identification of Y chromosome sequences in the human and *Drosophila* genomes. Genome Res. 23(11):1894–1907. https://doi.org/10.1101/gr.156034.113

Carvalho AB, Dobo BA, Vibranovski MD, Clark AG. 2001. Identification of five new genes on the Y chromosome of *Drosophila melanogaster*. Proc Natl Acad Sci U S A. 98(23):13225–13230. https://doi.org/10.1073/pnas.231484998

Carvalho AB, Kim BY, Uno F. 2025. Strong sequencing bias in Nanopore and PacBio prevents assembly of *Drosophila melanogaster* Y-linked genes. https://doi.org/10.1101/2025.02.23.639762

Carvalho AB, Lazzaro BP, Clark AG. 2000. Y chromosomal fertility factors *kl-2* and *kl-3* of *Drosophila melanogaster* encode dynein heavy chain polypeptides. Proceedings of the National Academy of Sciences. 97(24):13239–13244. https://doi.org/10.1073/pnas.230438397

Chang CH et al. 2019. Islands of retroelements are major components of *Drosophila* centromeres. PLoS Biol. 17(5). https://doi.org/10.1371/journal.pbio.3000241

Chang CH, Larracuente AM. 2019. Heterochromatin-enriched assemblies reveal the sequence and organization of the *Drosophila melanogaster* Y Chromosome. Genetics. 211(1):333–348. https://doi.org/10.1534/genetics.118.301765

Gatti M, Pimpinelli S. 1983. Cytological and genetic analysis of the Y chromosome of *Drosophila melanogaster*. Chromosoma. 88(5):349–373. https://doi.org/10.1007/BF00285858

Gepner J, Hays TS. 1993. A fertility region on the Y chromosome of *Drosophila melanogaster* encodes a dynein microtubule motor. Proceedings of the National Academy of Sciences. 90(23):11132–11136. https://doi.org/10.1073/pnas.90.23.11132

Hoskins RA et al. 2007. Sequence Finishing and Mapping of *Drosophila melanogaster* Heterochromatin. Science (1979). 316(5831):1625–1628. https://doi.org/10.1126/science.1139816

Hoskins RA et al. 2015. The Release 6 reference sequence of the *Drosophila melanogaster* genome. Genome Res. 25(3):445–458. https://doi.org/10.1101/gr.185579.114

Jia H et al. 2024. Low-input PacBio sequencing generates high-quality individual fly genomes and characterizes mutational processes. Nat Commun. 15(1). https://doi.org/10.1038/s41467-024-49992-6

Kennison JA. 1981. The Genetic and Cytological Organization of the *Y* Chromosome of *Drosophila melanogaster*. Genetics. 98(3):529–548. https://doi.org/10.1093/genetics/98.3.529

Kim BY et al. 2024. Single-fly genome assemblies fill major phylogenomic gaps across the Drosophilidae Tree of Life Jiggins CD, editor. PLoS Biol. 22(7):e3002697. https://doi.org/10.1371/journal.pbio.3002697

Krsticevic FJ, Schrago CG, Carvalho AB. 2015. Long-Read Single Molecule Sequencing to Resolve Tandem Gene Copies: The *Mst77Y* Region on the *Drosophila melanogaster* Y Chromosome. G3 Genes|Genomes|Genetics. 5(6):1145–1150. https://doi.org/10.1534/g3.115.017277

Livak KJ. 1990. Detailed structure of the *Drosophila melanogaster* stellate genes and their transcripts. Genetics. 124(2):303–316 https://academic.oup.com/genetics/article/124/2/303/6000006. https://doi.org/10.1093/genetics/124.2.303

Russell SR, Kaiser K. 1993. *Drosophila melanogaster* male germ line-specific transcripts with autosomal and Y-linked genes. Genetics. 134(1):293–308. https://doi.org/10.1093/genetics/134.1.293

Tobler R, Nolte V, Schlötterer C. 2017. High rate of translocation-based gene birth on the *Drosophila* Y chromosome. Proceedings of the National Academy of Sciences. 114(44):11721–11726. https://doi.org/10.1073/pnas.1706502114

Vibranovski MD, Koerich LB, Carvalho AB. 2008. Two New Y-Linked Genes in *Drosophila melanogaster*. Genetics. 179(4):2325–2327. https://doi.org/10.1534/genetics.108.086819
